# Maternal and Child Health Care Service Disruptions and Recovery in Mozambique After Cyclone Idai: An Uncontrolled Interrupted Time Series Analysis

**DOI:** 10.9745/GHSP-D-21-00796

**Published:** 2022-09-15

**Authors:** Quinhas Fernandes, Orvalho Augusto, Sérgio Chicumbe, Laura Anselmi, Bradley H. Wagenaar, Rosa Marlene, Sãozinha Agostinho, Sarah Gimbel, James Pfeiffer, Celso Inguane, Dorlim Moiana Uetela, Jonny Crocker, Isaías Ramiro, Benigna Matsinhe, Stélio Tembe, Naziat Carimo, Stephen Gloyd, Ivan Manhiça, Esperança Tavede, Priscilla Felimone, Kenneth Sherr

**Affiliations:** a National Directorate of Public Health, Ministry of Health, Mozambique.; b Department of Global Health, University of Washington, Seattle, WA, USA.; c Eduardo Mondlane University, Maputo, Mozambique.; d Instituto Nacional de Saúde, Ministry of Health, Mozambique.; e Centre for Primary Care and Health Services Research, University of Manchester, Manchester, United Kingdom.; f Department of Epidemiology, University of Washington, Seattle, WA, USA.; g Mozambique Permanent Mission, Geneva, Switzerland.; h National Directorate of Planning and Cooperation, Ministry of Health, Mozambique.; i Department of Child, Family, and Population Health Nursing, School of Nursing, University of Washington, Seattle, WA, USA.; j Department of Anthropology, University of Washington, Seattle, WA, USA.; k Comité para a Saúde de Moçambique, Maputo, Mozambique.; l Provincial Social Affairs Services, Manica, Mozambique.; m Provincial Social Affairs Services, Sofala, Mozambique.; n Department of Industrial & Systems Engineering, University of Washington.

## Abstract

**Introduction:**

Climate change-related extreme weather events have increased in frequency and intensity, threatening people’s health, particularly in places with weak health systems. In March 2019, Cyclone Idai devastated Mozambique’s central region, causing infrastructure destruction, population displacement, and death. We assessed the impact of Idai on maternal and child health services and recovery in the Sofala and Manica provinces.

**Methods:**

Using monthly district-level routine data from November 2016 to March 2020, we performed an uncontrolled interrupted time series analysis to assess changes in 10 maternal and child health indicators in all 25 districts before and after Idai. We applied a Bayesian hierarchical negative binomial model with district-level random intercepts and slopes to estimate Idai-related service disruptions and recovery.

**Results:**

Of the 4.44 million people in Sofala and Manica, 1.83 (41.2%) million were affected. Buzi, Nhamatanda, and Dondo (all in Sofala province) had the highest proportion of people affected. After Idai, all 10 indicators showed an abrupt substantial decrease. First antenatal care visits per 100,000 women of reproductive age decreased by 23% (95% confidence interval [CI]=0.62, 0.96) in March and 11% (95% CI=0.75, 1.07) in April. BCG vaccinations per 1,000 children under age 5 years declined by 21% (95% CI=0.69, 0.90) and measles vaccinations decreased by 25% (95% CI=0.64, 0.87) in March and remained similar in April. Within 3 months post-cyclone, almost all districts recovered to pre-Idai levels, including Buzi, which showed a 22% and 13% relative increase in the number of first antenatal care visits and BCG, respectively.

**Conclusion:**

We found substantial health service disruptions immediately after Idai, with greater impact in the most affected districts. The findings suggest impressive recovery post-Idai, emphasizing the need to build resilient health systems to ensure quality health care during and after natural disasters.

## Abstract (precis)

Timely and relevant information is vital to help identify and track areas of improvement after extreme weather events and during emergencies to prioritize limited resources. Routine data can provide useful evidence of health system performance during and after natural disasters, contributing to an effective and efficient response.

## Teaser

**Key Findings:**

- Substantial disruptions in service delivery were observed immediately after Cyclone Idai, with severely affected districts showing greater disruptions. Overall, first antenatal care visits, postpartum visits within 3–7 days, new family planning users, measles vaccinations, first at-risk children’s consultations, and fully immunized children under age 1 were the most affected indicators.
- Within 3 months of the cyclone, we observed the recovery, across all districts, of indicators to levels equal to or higher than pre-Idai. This quick recovery showcases Mozambique’s health system resilience, particularly in the central region.
- Implementation research methods combined with high-quality routine data provide relevant evidence to support policy making and strategic decisions, particularly in scenarios of extreme weather events such as Cyclone Idai; therefore, they should be prioritized.

**Key Implication:**

- While maternal and child health care delivery is negatively impacted by extreme weather events, lessons learned from the recovery process can help strengthen health systems. Policy makers should prioritize routine health information data as a valuable tool for tracking health system resilience.

Resumo em português no final do artigo.

## INTRODUCTION

Increasingly, extreme weather events such as floods, storms, and cyclones present a permanent threat to health systems and individual health outcomes, particularly in low- and middle-income countries.^1^ Worldwide, roughly 7,300 natural disasters occurred between 2000 and 2019, resulting in 1.2 million deaths (yearly average of 35,000) and an economic loss of US$2.97 trillion.^1^^,^^2^ Floods and storms are the most frequent events, accounting for 44% and 28% of natural disasters, respectively.^1^ Exposure to extreme weather events leads to immediate service disruptions, increased burden of infectious and non-infectious diseases, and poor long-term health outcomes.^3^^–^^8^ A systematic review from 2012 found a 47% to 50% increase in the population mortality at all ages and a 40% increase in mental health disorders among individuals older than age 14 years in the first year after a severe flood episode.^2^^,^^9^^–^^11^

After being exposed to an extreme weather event, older adults are 2.1 times more likely than younger people to experience post-traumatic stress and 1.7 times more likely to develop a subsequent adjustment disorder^12^; pregnant women are at increased risk of pre-term delivery and low birth weight^13^^,^^14^; and children may experience an 18% increase in diarrhea and a 15% increase in acute respiratory infections.^15^ Regardless of the magnitude of the event, women and children are most vulnerable to disruptions in health care (e.g., immunization and maternal and child health services). Antenatal care (ANC) visits, institutional deliveries, and postpartum care visits for both mothers and newborns are significantly reduced in areas recurrently affected by floods, as suggested by a study conducted in Bangladesh.^16^

The impact of extreme weather events on public infrastructure is expected, leading to reduced accessibility, availability, and quality of health care services, particularly with higher-magnitude events.^4^^,^^6^ Power outages frequently occur and are consistently associated with poor patient outcomes, as they affect quality of care for both chronic and acute conditions.^17^ Regardless of severity, resilient health systems are expected to maintain essential services while responding to initial shocks and recover quickly.^18^ The speed of recovery is related to the shock’s magnitude, its characteristics, the population’s baseline vulnerability, and the health system’s level of resilience.^1^ Health system resilience is defined as the capacity to absorb external shocks and adequately and promptly adjust to respond effectively, while maintaining all essential functions, including recovering any observed losses.^18^^–^^21^

Health system resilience is the capacity to absorb external shocks, adequately and promptly adjust to respond effectively, and maintain all essential functions.

Because of its geographic location, Mozambique is highly vulnerable to floods and cyclones. On March 14, 2019, category 4 Cyclone Idai hit Mozambique’s central region (Zambezia, Tete, Sofala, and Manica Provinces), directly affecting 2.1 million people and causing 603 deaths, 1,641 injuries, and the displacement of 400,000 people.^22^^,^^23^ Even though neighboring provinces (Tete and Zambézia) experienced high-speed winds and rains, the cyclone magnitude there was substantially lower and resulted in fewer fatalities, less destruction, and fewer health service disruptions were reported. No meaningful impacts from Idai were observed in any other provinces.

With the disruption of essential services (e.g., water, electricity, and communications) and significant damage to public infrastructure (including 90 health facilities and 3,145 health workers’ homes, particularly in Manica and Sofala), the effects of Idai exacerbated ongoing challenges in sanitation, water supply, and food security in these 2 provinces.^24^^,^^25^ One month after the cyclone, a cholera outbreak affected 4 districts in Sofala Province (Beira City, Dondo, Nhamatanda, and Buzi), with 6,768 reported cases and 8 deaths (0.12% case fatality).^22^ Given the extent of the destruction, the budget needed to rebuild the health infrastructure was estimated at US$202 million over 5 years, with the first half needed in the first year (unpublished data). Domestic and international solidarity in the aftermath of Idai was impressive. The national government, bilateral organizations, and multilateral international institutions played a critical role in saving lives, bridging gaps in health service disruptions, and mobilizing resources for a comprehensive response plan to address immediate and long-term needs.

There is limited evidence on health service continuity and the speed of recovery during and after an extreme weather event, especially in low- and middle-income countries. Routine health information system (RHIS) data, which are frequently disparaged due to quality issues, might be the best source to describe health service impacts with high resolution, granularity, and availability; therefore, these systems provide a critical opportunity to understand how health systems adjust to external shocks and guide policy makers’ decisions. However, they can also be affected during external shocks, which may impede efforts to distinguish whether disruptions reflect service discontinuity or simply a lack of data.

In the 3 years since Idai, Mozambique has made significant progress toward rebuilding its health infrastructure and ensuring the provision of primary health care services. Mozambique’s experience with Idai has offered a unique opportunity to understand the nature of health service disruptions after an extreme weather event and the speed of the health system’s recovery to pre-disaster levels. In this study, we aimed to assess Idai’s impact on district-level maternal and child health care services in the 2 most affected provinces (Sofala and Manica), as well as evaluate the health system’s recovery. We also intend to demonstrate the relevance of frequently overlooked RHIS data and propose a method to assess service disruptions and inform emergency response and preparedness plans. No other study has comprehensively investigated the effects of Idai on immediate health service utilization or Mozambique’s recovery process. Furthermore, to the best of our knowledge, no other study has investigated health system recovery after an extreme weather event by applying the methods used in this study.

Mozambique’s experience with Idai has offered a unique opportunity to understand the nature of health service disruptions after an extreme weather event and the speed of the health system’s recovery to pre-disaster levels.

## METHODS

### Study Design

Using a quasi-experimental design, we performed an uncontrolled interrupted time series analysis to assess monthly changes from November 2016 to March 2020 in 10 selected indicators.^26^^–^^29^ These indicators covered the continuum of maternal and child health service delivery in 25 districts across 2 provinces (Manica and Sofala) before and after Cyclone Idai.

### Setting

We assumed 10% as the cut-off value for the percentage of people affected by Idai. Manica and Sofala provinces were selected due to the percentage of people (41.2%) affected in those provinces, a proxy measure of Idai’s impact. Zambezia and Tete provinces were excluded because less than 5% of the total province population was affected.

**FIGURE 1:**
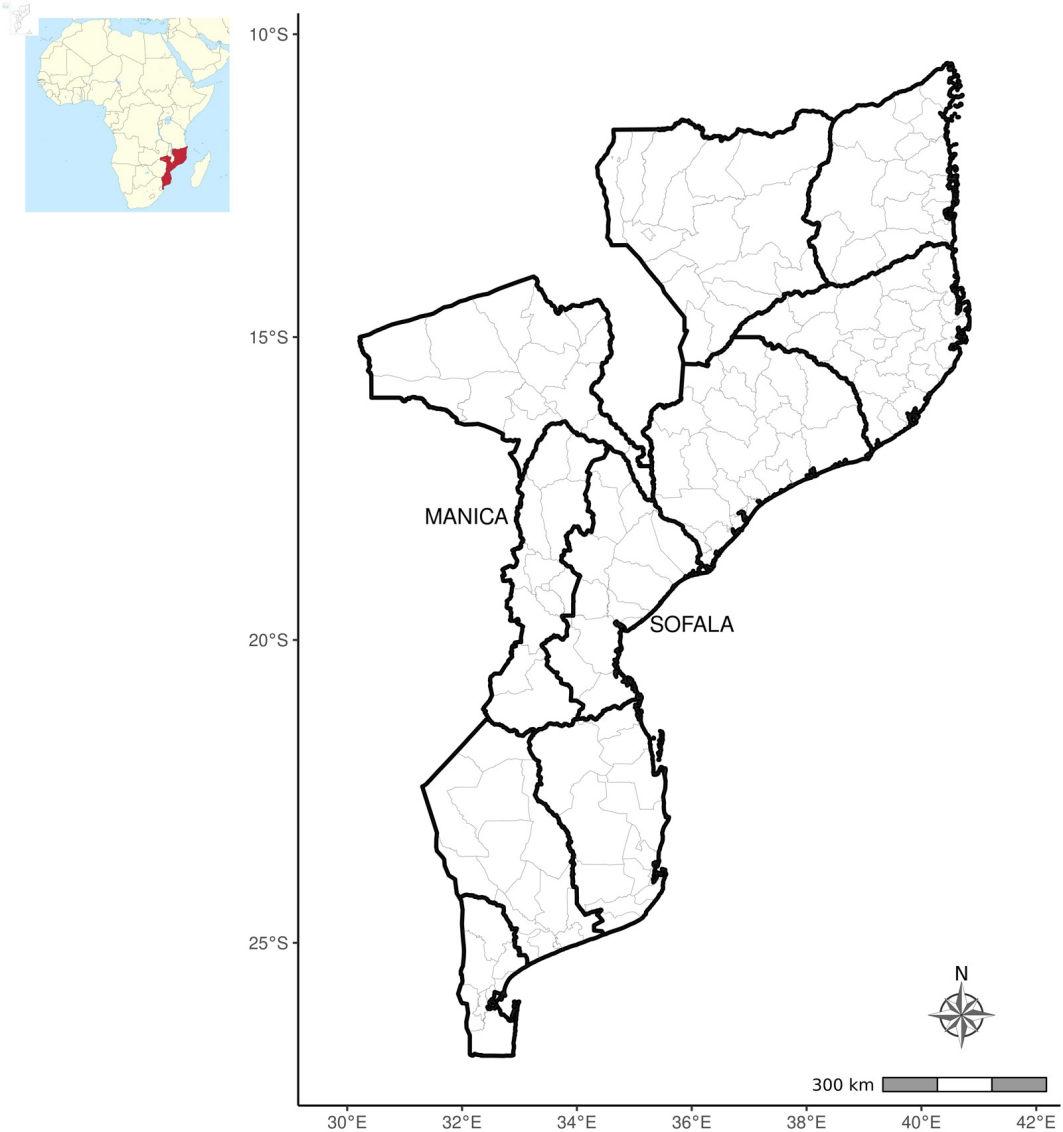
Map of Sofala and Manica Provinces in Mozambique

Sofala and Manica are neighboring provinces located in the central region of Mozambique (Figure 1). Sofala is situated along the coast of the Indian Ocean, whereas Manica borders Zimbabwe to the west. Sofala is among the poorest of Mozambique’s 11 provinces. In 2017, Sofala’s population was 2.3 million, of which 60% were living in rural areas; Manica’s population was 1.9 million, with 66% living in rural areas. In both provinces, the under-5 mortality rate was higher than 100 deaths per 1,000 live births in 2011.^24^^,^^30^ For administrative purposes, the cities of Beira and Chimoio are considered districts.

### Maternal and Child Health Service Delivery Outcomes

Ten indicators available through the RHIS were selected to reflect a range of maternal and child services at the primary health care level. Together they were used to assess Idai’s impact on service utilization. The following indicators were assessed using monthly aggregated counts at the district level: (1) first antenatal care visits; (2) women completing at least 4 doses of intermittent preventive treatment for malaria during pregnancy (IPTp4); (3) institutional deliveries; (4) postpartum care visits within 3 to 7 days after delivery; (5) new users of modern contraceptives; (6) bacillus Calmette-Guerin (BCG) vaccinations; (7) diphtheria, pertussis, tetanus, and *Haemophilus influenzae* type b (DPT-Hib3) vaccinations; (8) measles vaccinations; (9) fully immunized children under age 1; and (10) first consultations for pediatric at-risk services.

### Cyclone Idai

Cyclone Idai was characterized by high-speed winds reaching more than 118 miles (190 kilometers) per hour and heavy rains (200 mm per day). The cyclone led to destruction and flooding in districts and communities surrounding Buzi, a prominent regional river. On its trajectory, Idai inflicted the most damage to Sofala and Manica provinces. Within the provinces, 7 districts (4 in Sofala and 3 in Manica) experienced the highest impact. Beira City, where Idai made landfall in Sofala province, was mostly affected by high-speed winds. However, Buzi District, also in Sofala, suffered from both high-speed winds and dramatic floods that covered almost the entire district.

Given the heterogeneity in the levels of destruction, we characterized Idai’s severity based on the proportion of people affected in each district (estimated as the number of people affected among the total district population). We defined this as the “resident population whose homes were affected by shelter damage and have not left the assessed locality.”^31^ We created 3 strata of districts: I) least affected, II) moderately affected, and III) highly affected. All districts with no affected people were included in strata I. We used the median (of the proportion of affected people) to separate strata II and strata III (Table 1).

**TABLE 1.** Population and Level of Cyclone Idai Destruction in 25 Districts of Manica and Sofala Provinces, Mozambique, 2019

	**2019 Population** ^a^			**Cyclone Idai Impact** ^b^	
**Districts**	**Total Population**	**Women Aged 15–49 Years, %**	**Children Aged Younger Than 5 Years, %**	**Affected People, No. (%)**	**Strata**^c^
Manica Province	2,056,037	30.2	17.9	414,977 (20.2)	
Bárue	205,756	30.6	17.6	-	I
Chimoio City	417,954	31.8	16.0	1,603 (0.4)	II
Gondola	212,930	30.0	17.7	104,528 (49.1)	III
Guro	105,906	29.6	18.4	-	I
Macate	92,059	28.7	18.4	51,284 (55.7)	III
Machaze	137,857	31.9	18.3	21,576 (15.7)	II
Macossa	50,294	29.7	18.4	-	I
Manica	241,122	30.3	17.2	10,234 (4.2)	II
Mossurize	222,078	31.0	18.3	51,068 (23.0)	II
Sussundenga	185,790	30.3	17.8	139,889 (75.3)	III
Tambarra	58,324	29.9	18.4	-	I
Vanduzi	125,967	29.0	18.4	34,795 (27.6)	II
Sofala Province	2,388,902	30.6	16.8	1,416,690 (59.3)	
Búzi	191,693	30.7	17.1	247,193 (129.0)	III
Caia	171,334	30.5	16.9	-	I
Chemba	89,784	30.5	17.4	-	I
Cheringoma	62,586	30.7	16.3	-	I
Chibabava	145,778	31.5	17.4	41,204 (28.3)	II
Beira City	651,313	31.7	14.6	575,077 (88.3)	III
Dondo	210,742	30.5	16.0	211,266 (100.2)	III
Gorongosa	191,565	30.6	16.8	-	I
Machanga	60,223	30.8	17.4	20,004 (33.2)	II
Maríngue	102,833	30.8	17.4	-	I
Marromeu	167,791	30.3	16.3	-	I
Muanza	41,179	28.7	17.5	34,202 (83.1)	III
Nhamatanda	302,081	30.0	16.9	287,744 (95.3)	III
Total	4,444,939	30.4	17.3	1,831,667 (41.2)	** **

a Projected population based on National Institute of Statistics (INE) 2007 Census.^32^

b National Institute for Disaster Management (INGD) reports.^29^

c Based on the number of people affected (percentage) after Cyclone Idai, strata I districts were the least affected, strata II were medium affected, and strata III were highly affected.

### Data Collection and Processing

Data on selected indicators were sourced from the health information system (Sistema de Informação de Saúde para Monitoria e Avaliação, or SISMA), based on the District Health Information Management System 2 (DHIS-2), from November 2016 to March 2020. Population data (including the total number of women of reproductive age and children under 5) were sourced from the 2007 Population and Housing Census, using district-level projections for 2017. After 2017, Manica District was divided into 2 districts (Manica and Vanduzi), and Gondola District was divided into Gondola and Macate districts. Data from the 2017 census were used to distribute the projected population of Manica and Gondola districts proportionately into these new districts.

We collected and analyzed data for 25 districts (13 in Sofala Province and 12 in Manica Province) in Mozambique over a period of 42 consecutive months from November 2016 to March 2020. Of the 25 districts, we classified 10 as least affected (strata I), 7 as medium affected (strata II), and 8 as highly affected (strata III) by Cyclone Idai, based on the number of people affected.^31^

### Statistical Analysis

We explored the data to assess completion and the presence of potential outliers to inform the analysis. Furthermore, we assessed district-specific time series plots to identify the parametrization of the models. For district comparisons, we used descriptive statistics (mean, standard deviation, coefficient of variation [CV], minimum, and maximum). We modeled the monthly counts of service provision, accounting for yearly estimated population and potential overdispersion through a negative binomial regression with district-specific random effects (intercepts and slopes) using an equation (Box).

BOX **Model Used to Calculate Monthly Counts for Service Provision After Cyclone Idai**

$$\begin{array}{c} \text{log}\left(count_{dt}\right)=\left(\beta_{0}+b_{0}^{*d}\right)\\ +\;\left(\beta_{post}+b_{post}^{*d}\right)\cdot I\left(time\geq March2019\right)\\ +\;\left(\beta_{March2019}+b_{March2019}^{*d}\right)\cdot I\left(time=March2019\right)+\left(\beta_{April2019}+b_{April2019}^{*d}\right)\cdot I\left(time=April2019\right)\\ +\;\left(\beta_{pre}+b_{pre}^{*d}\right)\cdot time+\left(\beta_{postslope}+b_{postslope}^{*d}\right)\cdot time^{*}\\ +\;Season\left(time\right)+1\cdot \;\text{log}\;\left(Population_{dt}\right) \end{array}$$

where *count* is the count of services delivered from a district *d* at time *t*. The subscript *t* and the variable *time* indicate time in months since November 2016, and for the variable time* since March 2019 (taking value zero otherwise). The *I(time ≥ March2019)*, *I(time = March2019)*, and *I(time = April2019)* represent dummy indicators for time past March 2019, only March 2019, and only April 2019, respectively. Their coefficients capture the overall immediate impact post-Cyclone Idai (β_post_) on the health system, and specific March (β_March2019_) and April 2019 (β_April2019_) impacts. *Season(time)* represents a function of time to capture seasonal trends, here chosen as 11 dummy indicators for each month with January as reference. The yearly projected population (women ages 15 to 49 or children under 5) was added as an offset.

β_0_ is the intercept (overall log-mean count on November 2016), β_pre_ is the overall monthly change in log counts for all 25 districts before the cyclone, β_post_ is the immediate overall change in log counts for all districts since March 2019 (affecting the overall post-Cyclone Idai period), β_March2019_ and β_April2019_ are March- and April 2019-specific deviations from the overall β_post_, β_postslope_ is the overall monthly change in log counts during the post-cyclone period, and b* coefficients are district-specific deviations from the respective β coefficient. The above model works on counts per population; therefore, the exponentiated coefficients are to be interpreted as relative changes in the count services per population. So immediately after Idai, there was e^βpost^, e^βpost + βMach2019^ and e^βpost + βApril2019^ for overall months, specific to March 2019, and specific to April 2019, respectively, associated level change in count services per population. We focus on these 2 months as they represent the period of greatest impact.

We computed the relative losses as the ratio between the observed counts for Idai and the expected counts for a district in a particular month since March 2019. The predicted counts are estimated from the model above with the on and off Idai scenarios set by the indicators I() terms as 1 or 0, respectively. The calculations are done at the district level. We assessed the relative loss by aggregating districts into their respective strata of cyclone damage.

The above equation is estimated as generalized linear mixed model (GLMM)^32^ with negative binomial family and log link for each of the 10 service provision indicators. GLMMs are an extension of GLM to include random effects to address the nested and clustered nature of the data (e.g., 1 district has monthly counts making 42 observations). The GLMMs can be estimated through Maximum-Likelihood (ML), restricted ML (REML), and Bayesian approaches.^33^ Due to numeric estimation challenges (multiple random effects and issues of estimation convergence) and less bias on the variance of the random effects, we chose to use the Bayesian approach.^34^ All regression models were estimated using Stan programming language through the brms package in RStudio version 3.6.3 (RStudio).^35^^,^^36^ As priors, we chose for fixed effects coefficients univariate normal distribution with 0 mean and 100 variance, for each standard deviation of the random effects a half Student-t with 3 degrees of freedom and scale parameter that is minimally 10, and for the negative binomial shape parameter a gamma distribution with 0.01 for shape and rate parameter. For correlations, the default priors were left unchanged. Four parallel chains were fit with 16,000 iterations, of which the first 1,000 were discarded as burn-in. We used Gelman-Rubin diagnostics (less than 1.02), trace plots, and autocorrelations to assess convergence, good mixing, and iteration autocorrelation. We applied a thin of 5, resulting in 12,000 iterations remaining for the posterior estimation. These were used to estimate the relative loss of service delivery in March, April, and May 2019 and to assess the immediate losses due to the cyclone and the subsequent overall recovery. We focused the analysis on 3 months to capture the first month of returning to pre-Idai levels. The exponentiated parameters β_jump_ and β_post_ indicate the overall relative changes across 25 districts in the months after Cyclone Idai.

### Ethics Approval

We used monthly district-level aggregated data, with approval from the Ministry of Health. We extracted data from the health information system devoid of individual-level identifiers.

## RESULTS

Of the 4.44 million people living in Sofala and Manica provinces, 41.2% (1.83 million) were affected by the cyclone. Overall, the greatest impact was observed in Buzi, Dondo, and Nhamatanda, where almost all district inhabitants were affected.^31^^,^^37^ Table 1 shows the district-specific population and the proportion of affected people.

### District Characteristics Before Cyclone Idai

In 2017, for each of the 10 selected indicators, the cities of Beira and Chimoio (provincial capitals) had substantially higher average monthly counts than their respective provinces. The average number of first ANC visits was 847 (CV: 0.51) in Manica and 813 (CV: 0.61) in Sofala. Across the 25 districts, the monthly average number of first ANC visits ranged from 304 (CV: 0.05) in Machanga to 2,015 (CV: 0.12) in Beira City. The monthly average number of institutional deliveries was similar in both provinces: 562 (CV: 0.73) in Sofala and 558 (CV: 0.53) in Manica. The monthly mean number of new users of modern contraceptives was higher in Manica (2,150; CV: 1.24) than in Sofala (1,663; CV:1.12). In Manica, the monthly average number of children who completed all vaccines within the first year of life fluctuated from 192 (CV: 0.31) in Tambara to 1,096 (CV: 0.18) in Chimoio City, while in Sofala, it ranged from 124 (CV:0.31) in Muanza to 1,201 (CV:0.22) in Beira City. Compared to the number of institutional deliveries, the mean number of postpartum visits was lower than expected—less than one-sixth of the mean institutional deliveries for both provinces. Table 2 shows the monthly average counts for each indicator per district.

**TABLE 2.** Monthly Averages for Each Study Indicator in 25 Districts of Manica and Sofala Provinces, Mozambique, 2017

**District/Province**	**First ANC Visits**	**IPTp4**	**Institutional Deliveries**	**New FP User**	**Measles Vaccination**	**BCG Vaccination**	**DPT-Hib3** **Vaccination**	**Fully Immunized Children Under Age 1 Year**	**First At-Risk Children’s Consultation**	**Postpartum Care Visit (3–7 days)**
	**Mean (CV)** ^a^	**Mean (CV)** ^a^	**Mean (CV)** ^a^	**Mean (CV)** ^a^	**Mean (CV)** ^a^	**Mean (CV)** ^a^	**Mean (CV)** ^a^	**Mean (CV)** ^a^	**Mean (CV)** ^a^	**Mean (CV)** ^a^
Manica Province^b^	847 (0.51)	371 (0.68)	558 (0.53)	2,150 (1.24)	607 (0.57)	681 (0.67)	604 (0.56)	574 (0.64)	74 (0.75)	86 (1.06)
Bárue	952 (0.20)	290 (0.35)	675 (0.16)	3,953 (1.32)	764 (0.36)	901 (0.29)	801 (0.36)	779 (0.78)	101 (0.32)	71 (0.77)
Chimoio City	1,708 (0.17)	935 (0.19)	1,282 (0.07)	4,200 (0.65)	1,150 (0.16)	1,455 (0.11)	1,191 (0.10)	1,096 (0.18)	214 (0.11)	319 (0.27)
Gondola	1,064 (0.08)	482 (0.12)	660 (0.04)	3,215 (1.24)	610 (0.36)	688 (0.21)	580 (0.31)	586 (0.35)	72 (0.30)	117 (0.42)
Guro	514 (0.07)	324 (0.22)	347 (0.10)	985 (1.13)	351 (0.14)	389 (0.12)	355 (0.14)	323 (0.16)	46 (0.52)	100 (0.40)
Macate	598 (0.41)	327 (0.16)	369 (0.11)	1,217 (0.95)	436 (0.46)	462 (0.44)	435 (0.49)	426 (0.46)	46 (0.25)	35 (1.12)
Machaze	612 (0.11)	101 (0.39)	420 (0.10)	1,317 (0.74)	548 (0.50)	682 (0.81)	528 (0.34)	514 (0.44)	56 (0.26)	37 (0.49)
Macossa	222 (0.15)	84 (0.31)	166 (0.05)	598 (0.77)	184 (0.39)	167 (0.20)	166 (0.15)	159 (0.45)	10 (0.81)	33 (1.56)
Manica	1,077 (0.08)	612 (0.12)	672 (0.08)	2,069 (0.66)	744 (0.16)	781 (0.13)	771 (0.10)	705 (0.19)	115 (0.11)	126 (0.26)
Mossurize	1,346 (0.12)	561 (0.13)	803 (0.04)	2,988 (0.41)	1,171 (0.22)	1,131 (0.69)	1,076 (0.25)	1,092 (0.22)	52 (0.41)	45 (1.25)
Sussundenga	953 (0.09)	260 (0.24)	601 (0.08)	2039 (1.04)	598 (0.16)	692 (0.18)	601 (0.17)	558 (0.16)	67 (0.38)	79 (0.47)
Tambarra	364 (0.31)	54 (0.18)	195 (0.15)	1,722 (1.81)	230 (0.25)	252 (0.23)	232 (0.20)	192 (0.31)	17 (0.22)	40 (0.47)
Vanduzi	713 (0.11)	371 (0.23)	510 (0.08)	1,494 (1.34)	504 (0.20)	576 (0.13)	506 (0.16)	460 (0.21)	47 (0.15)	12 (1.28)
Sofala Province^b^	813 (0.61)	289 (0.84)	562 (0.73)	1,663 (1.12)	579 (0.67)	653 (0.66)	597 (0.67)	493 (0.65)	125 (1.19)	67 (1.06)
Búzi	938 (0.12)	315 (0.15)	697 (0.09)	2,737 (0.87)	750 (0.27)	784 (0.13)	810 (0.27)	687 (0.22)	139 (0.27)	51 (0.92)
Caia	884 (0.07)	307 (0.14)	650 (0.07)	2,395 (1.34)	715 (0.51)	788 (0.35)	747 (0.51)	607 (0.45)	138 (0.26)	90 (0.43)
Chemba	445 (0.14)	41 (0.90)	275 (0.08)	1,099 (0.58)	286 (0.18)	341 (0.12)	296 (0.20)	243 (0.19)	33 (0.39)	23 (0.90)
Cheringoma	362 (0.09)	54 (0.26)	245 (0.08)	379 (1.01)	241 (0.37)	295 (0.30)	267 (0.33)	211 (0.37)	20 (0.45)	32 (0.35)
Chibabava	704 (0.09)	213 (0.21)	403 (0.13)	854 (0.87)	555 (0.19)	571 (0.11)	552 (0.16)	462 (0.15)	87 (0.21)	80 (0.19)
Beira City	2,015 (0.12)	872 (0.11)	1,657 (0.18)	3,889 (0.54)	1,479 (0.11)	1,668 (0.16)	1,510 (0.11)	1,201 (0.22)	574 (0.21)	197 (0.82)
Dondo	955 (0.14)	333 (0.16)	563 (0.12)	1961 (0.77)	603 (0.14)	694 (0.36)	637 (0.09)	565 (0.12)	145 (0.21)	98 (0.27)
Gorongosa	1,113 (0.17)	449 (0.30)	626 (0.11)	2,154 (0.32)	596 (0.11)	815 (0.16)	632 (0.12)	512 (0.10)	174 (0.47)	98 (0.27)
Machanga	304 (0.05)	136 (0.20)	242 (0.10)	522 (1.00)	240 (0.29)	248 (0.15)	228 (0.20)	224 (0.30)	43 (0.38)	22 (0.76)
Maríngue	526 (0.15)	119 (0.22)	308 (0.10)	858 (0.85)	321 (0.51)	354 (0.24)	311 (0.48)	218 (0.39)	40 (0.26)	40 (0.55)
Marromeu	759 (0.10)	250 (0.44)	493 (0.16)	2,167 (0.89)	557 (0.23)	642 (0.10)	604 (0.22)	504 (0.27)	60 (0.24)	36 (0.35)
Muanza	193 (0.15)	52 (0.23)	114 (0.11)	393 (1.10)	138 (0.36)	140 (0.24)	132 (0.21)	124 (0.31)	9 (0.53)	6 (0.71)
Nhamatanda	1,374 (0.09)	601 (0.13)	1,037 (0.10)	2,208 (1.05)	1,041 (0.25)	1,155 (0.17)	1,028 (0.25)	850 (0.22)	161 (0.15)	64 (0.77)
Total	829 (0.56)	329 (0.76)	560 (0.64)	1,896 (1.21)	592 (0.62)	667 (0.66)	600 (0.62)	532 (0.65)	101 (1.17)	76 (1.07)

Abbreviations: ANC, antenatal care visit; BCG, bacillus Calmette-Guerin; CV, coefficient of variation (ratio of standard deviation to the average); DPT-Hib3, diphtheria, pertussis, tetanus, and *Haemophilus influenzae* type b immunization; FP, family planning; IPTp4, at least 4 doses of intermittent preventive treatment prophylaxis.

a Means and CV for the districts in the province.

b The averages and the CV are computed per district for the months of 2017.

### Regression Results

In November 2016, across all 25 districts, the average number of pregnant women per 100,000 women of reproductive age (WRA) who had completed a first ANC visit was 934 (95% CI=867, 1,007); of those, an average of 310 (95% CI=246, 392) women per 100,000 WRA completed at least 4 doses of IPTp and another 601 (95% CI=556, 649) per 100,000 WRA had delivered in a health facility. The average number of new users of modern contraceptives was 2,531 per 100,000 WRA (95% CI=2,107, 3,050) in November 2016. In the same month, on average, 2,293 (95% CI=2,080, 2,524) children per 100,000 children younger than 1 year were vaccinated against BCG, and another 2,113 (95% CI=1909, 2,342) had been vaccinated against measles.

Between November 2016 and February 2019, the period before Cyclone Idai, all indicators—except first ANC visits and postpartum visits—showed consistent and positive trends, although with significant heterogeneity across districts. Institutional deliveries per 100,000 WRA showed a significant monthly increase of 0.5%; (95% CI=1.00, 1.01), leading to an annual increase of 5.78% between the study baseline and February 2019. Similarly, pregnant women who completed at least 4 doses of IPTp (1.3%: 95% CI=1.01, 1.02) and new users of modern contraceptives (1.3%: 95% CI=1.01, 1.02) revealed significant monthly increases, culminating with annual gains of 16.9% and 17.0%, respectively, in the same period. Immunization trends before Idai (November 2016 to February 2019) were positive, with monthly increases of 0.4% (95% CI=1.00, 1.01) for measles vaccinations and 0.3% (95% CI=1.00, 1.01) for DPT-Hib3 vaccinations, corresponding to yearly gains of 5.3% and 3.0%, respectively. Before Idai, only postpartum visits had a significant negative trend, with a monthly loss of 3.4% (95% CI=0.95, 0.98).

**FIGURE 2:**
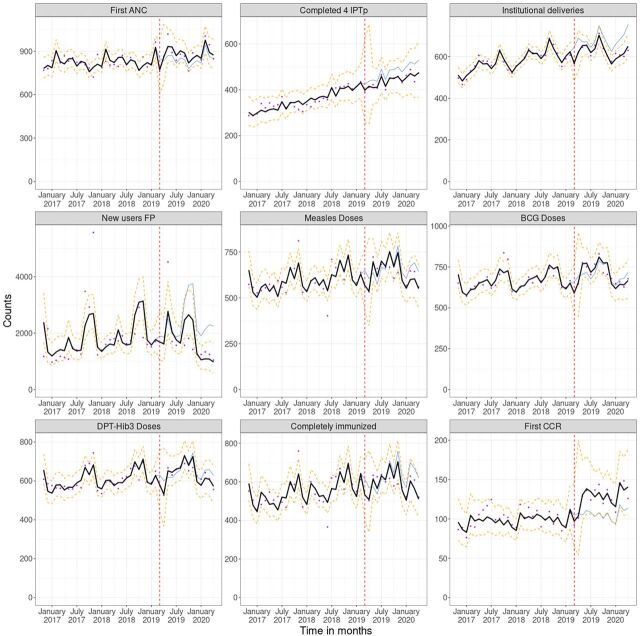
Average Counts for Service Delivery Indicators in 25 Districts Before and After Cyclone Idai in Sofala and Manica Provinces, Mozambique^a^ Abbreviations: ANC, antenatal care; BCG, bacillus Calmette-Guerin; CCR, child at risk consultation; DPT-Hib3, diphtheria, pertussis, tetanus, and Haemophilus influenzae type b; FP, family planning; IPTp4, at least 4 doses of intermittent preventive treatment prophylaxis. ^a^Cyclone Idai March 2019 (dashed red line); observed counts (dots); model expected under Idai (solid thick line) and its 95% confidence interval (dashed lines); counterfactual model expected without Idai (solid thin line).

When Cyclone Idai hit Mozambique in March 2019, all 10 district-level service delivery indicators showed a significant decline, which continued through April 2019 for most indicators. First ANC visits per 100,000 WRA decreased by 23.0% (95% CI=0.62, 0.96) in March 2019 and 11.0% (95% CI=0.75, 1.07) in April 2019. BCG and measles vaccinations per 1,000 children under 5 decreased by 21.0% (95% CI=0.69, 0.90) and 25.0% (95% CI=0.64, 0.87), respectively, and remained similar in April 2019. Statistically significant seasonal effects were seen across all indicators (except for IPTp4), but with different patterns; for example, every year, immunization services performed better in January, ANC services in April, and institutional deliveries in November. Table 3 shows the regression coefficients for each model. Figure 2 shows the overlap of the regression observed vs. fitted values.

**TABLE 3.** Exponentiated Regression Coefficient for Each Study Indicator Before and After Cyclone Idai, Manica and Sofala Provinces, Mozambique

**Indicator**	**Intercept** ^a^	**Post** ^b^	**Pre-Slope** ^c^	**Post-Slope Change** ^d^	**March 2019** ^e^	**April 2019** ^f^
	Exponentiated Regression Coefficient (95% CI)					
First ANC visit	934 (867, 1,007)	1.08 (1.02, 1.16)	0.998 (0.9965, 1.0011)	1.00 (0.99, 1.00)	0.77 (0.62, 0.96)	0.89 (0.75, 1.07)
IPTp4	310 (246, 392)	0.94 (0.85, 1.04)	1.013 (1.0080, 1.0183)	1.00 (0.98, 1.01)	0.90 (0.73, 1.11)	0.88 (0.69, 1.11)
Institutional delivery	601 (556, 649)	1.00 (0.96, 1.05)	1.005 (1.0024, 1.0069)	0.99 (0.98, 0.99)	0.87 (0.80, 0.94)	0.90 (0.84, 0.97)
New FP user	2,531 (2,107, 3,050)	1.34 (1.12, 1.60)	1.013 (1.0058, 1.0207)	0.91 (0.89, 0.93)	0.64 (0.48, 0.85)	0.69 (0.53, 0.92)
Measles vaccination	2,113 (1909, 2,342)	1.15 (1.06, 1.26)	1.004 (1.0016, 1.0069)	0.98 (0.97, 0.99)	0.75 (0.64, 0.87)	0.76 (0.62, 0.92)
BCG vaccination	2,293 (2,080, 2,524)	1.13 (1.06, 1.21)	1.001 (0.9991, 1.0035)	0.98 (0.98, 0.99)	0.79 (0.69, 0.90)	0.79 (0.65, 0.97)
DPT-Hib3 vaccination	2,121 (1910, 2,354)	1.09 (1.02, 1.17)	1.003 (1.0003, 1.0047)	0.99 (0.98, 0.99)	0.83 (0.73, 0.95)	0.77 (0.62, 0.95)
Fully immunized children under age 1 year	1,926 (1,722, 2,152)	1.13 (1.03, 1.24)	1.006 (1.0029, 1.0086)	0.98 (0.96, 0.99)	0.76 (0.65, 0.89)	0.79 (0.66, 0.94)
First at-risk children’s consultation	244 (187, 319)	1.27 (1.13, 1.42)	1.003 (0.9967, 1.0091)	1.01 (0.99, 1.02)	0.75 (0.63, 0.90)	0.73 (0.55, 0.95)
Postpartum visit within 3–7 days	103 (76, 139)	1.50 (1.13, 1.99)	0.966 (0.9538, 0.9781)	0.96 (0.92, 1.01)	0.57 (0.35, 0.94)	0.58 (0.37–0.92)

Abbreviations: ANC, antenatal care; BCG, bacillus Calmette-Guerin; DPT-Hib3, diphtheria, pertussis, tetanus, and Haemophilus influenzae type b; FP, family planning; IPTp4, at least 4 doses of intermittent preventive treatment prophylaxis.

a Intercept (e^β0^) is a model estimate of the count of service deliveries per 100,000 people in November 2016 (e.g., for “first ANC visit,” there were an estimated 934 first ANC visits per 100,000 women of reproductive age in November 2016).

b Post (e^βpost^) is the multiplicative change of the intercept since Cyclone Idai (e.g., compared to November 2016, there was an 8% increase in first ANC visits among women of reproductive health age in the post-Idai period [after March 2019]).

c Pre-slope (e^βpre^) is the monthly multiplicative increase in the ratio of indicator count to 100,000 people before Cyclone Idai (e.g., there was a 0.2% relative decrease in first ANC visits in women of reproductive health age per month).

d Post-slope (e^βpostslope^) change is the multiplicative change in the pre-slope coefficient (e.g., after Cyclone Idai, the monthly trend in first ANC visits was unchanged).

e March 2019 is the specific change relative to the post-level (e^βMarch2019^) change. This is a multiplicative deviation from the overall post-Idai level in March 2019 (e.g., during the month when Cyclone Idai occurred, the ratio of first ANC visits to the population of women of reproductive health age was 23% lower than during the entire post-Idai period).

f April 2019 is the specific change relative to the post-level (e^βApril2019^) change. This is a multiplicative deviation from the overall post-Idai level in April 2019 (e.g., during the month following Cyclone Idai, the ratio of first ANC visits to the population of women of reproductive health age was 11% lower relative to the entire post-Idai period).

When Cyclone Idai hit Mozambique in March 2019, all 10 district-level service delivery indicators showed a significant decline, which continued through April 2019 for most indicators.

### Relative Losses

Compared to model estimates without Cyclone Idai, all maternal health service delivery indicators showed immediate relative losses in March 2019 followed by recovery to levels before Idai in May (except IPTp4) and maintained these levels for the observation period. However, there was substantial variability across districts, with the most substantial losses observed in strata III districts. The overall immediate relative loss for first ANC visits was 11.0% in March (95% CI=0.72, 1.11), with 17% in Sofala and 5.0% in Manica. The following 3 districts showed the most significant relative losses in first ANC visits: 90.0% in Buzi (95% CI=0.07, 0.14), 28.0% in Machanga (95% CI=0.56, 0.93), and 23.0% in Tambara (95% CI=0.60, 0.99). Three months later (May 2019), almost all districts had recovered to pre-Idai levels, including Buzi, which showed a 22.0% (95% CI=1.05, 1.43) significant relative increase in first ANC visits. Overall, the number of new contraceptive users showed a relative decline of 14.0% (95% CI=0.56, 1.34), with 15.0% in Sofala and 13.0% in Manica; however, this was not significant. All districts recovered by May 2019. Regarding institutional deliveries, Manica showed a 9.0% (95% CI=0.78, 1.06) reduction in March 2019, while Sofala had a 15.0% (95% CI=0.73, 0.99) relative decrease.

Child health service delivery indicators showed similar results during the post-cyclone period, with service disruptions in March 2019, recovery to pre-Idai levels by May, and substantial variability across districts. Overall BCG vaccines dropped 10.0% (95% CI=0.71, 1.14) in March 2019, with 6.0% in Manica and 14.0% in Sofala. Buzi lost the most, with a 48.0% (95% CI=0.34, 0.82) decrease; however, by May 2019, Buzi had returned to pre-Idai levels with a 13.0% (95% CI=0.98, 1.31) relative increase. Concerning the measles vaccine, Buzi had a relative loss of 38.0% (95% CI=0.36, 0.92) in March 2019. Table 4 presents model-based estimates for the relative losses by selected indicators. The Supplement provides province- and district-specific estimates for all relative losses for March, April, and May 2019.

**TABLE 4.** Model Estimates and Relative Losses for Each Study Indicator After Cyclone Idai, Manica and Sofala Provinces, Mozambique, 2019

**Indicator**	**Observed**			**Model Expected Without Cyclone Idai**			**Model Expected With Cyclone Idai**			**Relative Loss** ^a^		
	**Count**			**Count (CI)**			**Count (CI)**			**(CI)**		
	**March**	**April**	**May**	**March**	**April**	**May**	**March**	**April**	**May**	**March**	**April**	**May**
First ANC visit	761	865	1,019	850	836	872	771	862	933	0.89 (0.72, 1.11)	1.00 (0.80, 1.26)	1.08 (0.95, 1.23)
				(780, 927)	(764, 914)	(794, 958)	(602, 986)	(683, 1,086)	(826, 1,053)			
IPTp4	391	411	422	423 (348, 513)	437 (356, 536)	440 (356, 544)	397 (256, 617)	415 (251, 685)	415 (345, 500)	0.88 (0.61, 1.31)	0.88 (0.57, 1.38)	0.94 (0.79, 1.11)
Institutional delivery	567	621	645	645 (598, 694)	689 (637, 744)	669 (617, 725)	569 (485, 666)	623 (536, 723)	657 (611, 706)	0.88 (0.75, 1.02)	0.90 (0.77, 1.05)	0.98 (0.90, 1.07)
New FP user	1,676	1,627	4,526	1921	1,879	2,520	1,694	1,615	2,808	0.86 (0.56, 1.34)	0.85 (0.56, 1.29)	1.12 (0.86, 1.45)
				(1,423, 2,594)	(1,375, 2,566)	(1,818, 3,493)	(1,000, 2,870)	(1,014, 2,575)	(2,092, 3,768)			
Measles vaccination	565	532	722	650 (569, 743)	606 (529, 695)	640 (556, 736)	566 (424, 756)	536 (350, 821)	701 (605, 813)	0.87 (0.67, 1.14)	0.87 (0.61, 1.29)	1.10 (0.97, 1.25)
BCG vaccination	591	646	775	659 (592, 733)	695 (623, 776)	706 (631, 789)	591 (457, 764)	655 (447, 960)	772 (690, 863)	0.90 (0.71, 1.14)	0.92 (0.67, 1.29)	1.10 (1.00, 1.21)
DPT-Hib3 vaccination	578	526	668	631 (567, 703)	602 (539, 673)	618 (551, 693)	579 (445, 754)	531 (368, 767)	654 (581, 736)	0.92 (0.72, 1.18)	0.87 (0.64, 1.23)	1.06 (0.96, 1.18)
Fully immunized children under age 1 year	532	503	634	615 (534, 709)	570 (493, 659)	575 (496, 666)	533 (395, 718)	508 (340, 759)	618 (537, 712)	0.86 (0.66, 1.12)	0.88 (0.63, 1.27)	1.07 (0.96, 1.20)
First at-risk children’s consultation	92	101	129	105 (81, 137)	108 (82, 143)	105 (78, 142)	96 (65, 142)	103 (54, 198)	130 (101, 168)	0.95 (0.72, 1.27)	0.97 (0.60, 1.65)	1.29 (1.04, 1.59)
Postpartum visit within 3–7 days	42	53	57	48 (22, 102)	53 (24, 117)	44 (19, 102)	44 (11, 174)	48 (13, 182)	60 (28, 130)	0.86 (0.40, 1.88)	0.84 (0.43, 1.64)	1.40 (0.95, 2.06)

Abbreviations: ANC, antenatal care; BCG, bacillus Calmette-Guerin; CI, confidence interval; DPT-Hib3, diphtheria, pertussis, tetanus, and Haemophilus influenzae type b; FP, family planning; IPTp4, at least 4 doses of intermittent preventive treatment prophylaxis.

a Relative loss is computed at the district level by dividing the Model Expected With Cyclone Idai scenario by the Model Expected Without Cyclone Idai scenario. Averages of these ratios are then computed for overall relative loss. The confidence intervals are computed from Markov Chain Monte-Carlo (MCMC) posterior realizations.

Two months after Idai, strata I districts were already returning to positive trends in all but 3 indicators (DPT-Hib3, IPTp4, and family planning); in contrast, highly affected strata III districts still had greater losses for all indicators except ANC visits and institutional deliveries.

**FIGURE 3:**
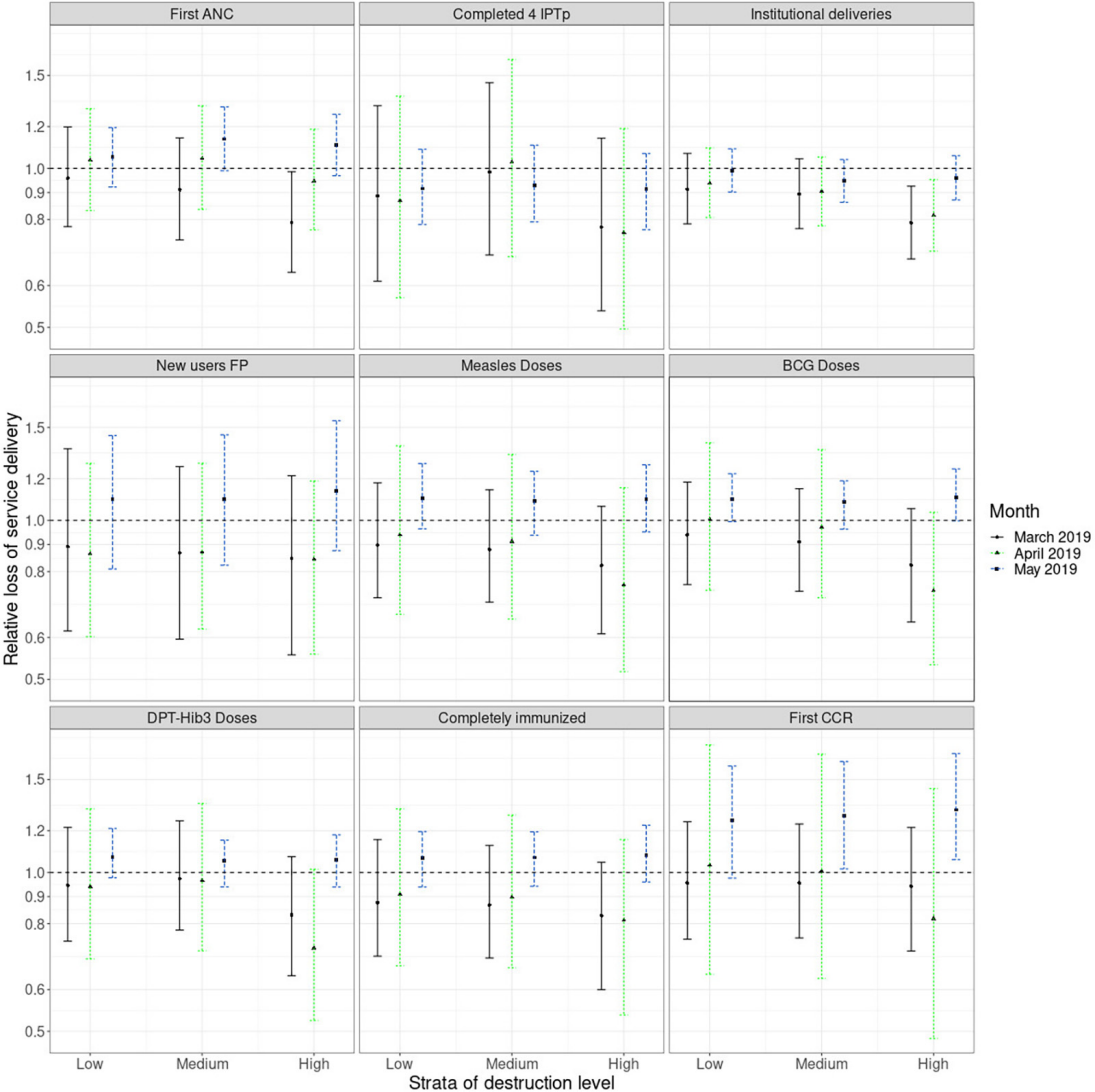
Relative Losses After Cyclone Idai, by Service Delivery Indicator and District-Level Destruction Strata, Sofala and Manica Provinces, Mozambique Abbreviations: ANC, antenatal care; BCG, bacillus Calmette-Guerin; CCR, child at risk consultation; DPT-Hib3, diphtheria, pertussis, tetanus, and Haemophilus influenzae type b; FP, family planning; IPTp4, at least 4 doses of intermittent preventive treatment prophylaxis.

Among strata III districts, the relative loss in institutional deliveries was 20.0% (95% CI=0.69, 0.94) in March 2019 and only 3.0% in May 2019. However, Buzi showed the most significant immediate relative loss in institutional deliveries, estimated at 55.0% (95% CI=0.36, 0.56) in March 2019. Despite its impressive recovery, Buzi still showed a 14.0% (95% CI=0.76, 0.97) relative loss in institutional deliveries in May 2019. The relative decline of BCG and measles vaccinations in March 2019 was 18.0% (95% CI=0.64, 1.06) and 18.0% (95% CI=61, 1.07), respectively. The 2 districts that showed the most significant projected relative losses in immunization were Buzi and Dondo. Model estimates for Buzi showed a loss of 48.0% (95% CI=0.34, 0.82) for BCG vaccinations and 38.0% (95% CI=0.36, 0.92) for measles vaccinations, and Dondo had a loss of 23.0% (95% CI=0.57, 0.95) and 22.0% (95% CI=0.56, 0.99), respectively, all in March 2019. Figure 3 shows the relative loss by strata.

## DISCUSSION

To determine disruptions to maternal and child health service delivery indicators after Cyclone Idai, we conducted an analysis that simultaneously accounted for annual population growth at the district level, seasonality, and historical trends using RHIS data. Overall, the results showed significant relative losses in all 10 selected indicators in the 2 months following Idai (March and April 2019), and a quick recovery within 3 months back to or higher than levels seen before the cyclone. Not surprisingly, strata III districts that were most seriously affected showed higher disruptions. These results corroborate previous findings from similar studies^16^^,^^38^^,^^39^ and are consistent with what could have been expected due to the massive destruction of the public health service infrastructure.^1^^,^^2^^,^^14^

To determine disruptions to maternal and child health service delivery indicators after Cyclone Idai, we conducted an analysis that simultaneously accounted for annual population growth at the district level, seasonality, and historical trends using RHIS data.

Even though returns to pre-Idai service delivery levels were observed by May 2019, this may not represent a full recovery, considering the need to recuperate losses from March and April. This is particularly true for indicators such as immunization, family planning, or antenatal care (for which care seeking could have been delayed), though not for other indicators that cannot be postponed (such as institutional deliveries). Therefore, studying the recovery process deserves special attention to the type of service disrupted; prospective studies may help researchers understand the full scope of recovery when service delivery trends return to levels before the shock. A study from Liberia quantified the cumulative losses not only to capture the return to expected trends but also to track whether losses from shock periods had been recovered.^26^ While this approach could help better describe the recovery, limitations persist—particularly when using aggregated data, as in this article. With aggregated data, it is difficult to disentangle whether specific groups recovered, particularly for chronically underperforming services, as higher than expected performance may not represent a full recovery but could instead be a consequence of increased demand due to targeting new groups or community mobilization activities.

The number of first consultations for at-risk child services was least affected in March and April 2019, particularly in strata I and II districts; however, this indicator showed the highest relative increase in May 2019 across all districts (Manica: 36%, Sofala: 22%), regardless of their level of destruction. This pattern might be due to increased demand for services for acute conditions, particularly malnutrition, which is predictable following an extreme weather event. Most at-risk child consultation visits are for children exposed to HIV or tuberculosis, or children being treated for malnutrition. In fact, 6 months after the cyclone, Sofala Province reported 600 cases of pellagra—a chronic B3 vitamin deficiency—after decades with no episodes, which signals a critical nutrition issue following Idai.^40^ Nutritional insecurity worsens after natural disasters, and vulnerable populations, particularly displaced people, face significant difficulties in securing daily meals due to possible increases in disruptions to travel, infrastructure destruction, and increased food prices, among others. Vulnerable groups often must rely on support from local and international organizations, which lacked resources or were delayed in some study areas.^41^

Several factors may have contributed to a quick health system recovery. Because the catastrophic impact of Idai captured widespread international attention, we assume that the aid received might have contributed to minimizing the initial shock and speeding up the recovery process. Domestic and international support contributed to swiftly addressing essential health system needs, including drugs, supplies, and materials. Furthermore, significant support existed to restore water supplies, electricity, and infrastructure rehabilitation—all critical service readiness determinants—though most support did not continue for an extended period and was focused on high-demand areas. Mozambique’s health sector has a well-established coordination mechanism with donors and implementing partners. Robust leadership and collaboration, together with massive individual solidarity, appear to have played an essential role in efficiently responding to Idai (at least during the initial shock period).^44^^,^^45^

Robust leadership and collaboration, together with massive individual solidarity, appear to have played an essential role in efficiently responding to Idai.

To the best of our knowledge, no other studies have focused on understanding how a health system recovers after the shock of an extreme weather event such as Cyclone Idai. Despite health system resilience being increasingly discussed in the field of global health, its definition, characteristics, and indicators of measurement are still subjects of debate.^42^ Notwithstanding, definitions of health system resilience have reached consensus in their inclusion of a health system’s ability to respond effectively to shocks and to maintain primary health system functions in the presence of external shocks or crises.^18^ Resilience is context-specific, adaptive, and builds on new learnings and knowledge translation into the health delivery system.^18^^,^^19^^,^^42^^–^^45^ The rapid and steady recovery seen across nearly all study indicators after the cyclone showcases critical signs of Mozambique’s health system resilience, particularly in Sofala and Manica. The substantial gains recorded within 3 months after the cyclone (by May 2019) reinforce the evidence for resilience —as the immediate international aid, in some cases, did not appear to continue beyond this timeframe.^46^ These findings are not surprising, since Mozambique has accumulated experience from previous cyclone responses (Dineo [2017], Hellen [2014], Funso [2012], and Leon-Eline [2000]), which has strengthened its preparedness to deal with shocks.

The rapid and steady recovery seen across nearly all study indicators after the cyclone showcases critical signs of Mozambique’s health system resilience, particularly in Sofala and Manica.

This study provides important lessons, particularly for low- and middle-income countries vulnerable to extreme weather events. First, the finding that highly affected areas had the most significant impact on services uptake reinforces the need to establish systems that quickly detect these areas during shock periods so that aid can be immediately channeled to support recovery plans. Second, even though we were able to observe a recovery to pre-Idai levels, this may not be enough since disruptions can be severe—as observed in Buzi—and a full recovery of the accumulated losses may take longer; therefore, robust monitoring mechanisms should be developed to continue tracking these losses. Third, external shocks can trigger acute conditions and create space for the emergence of related diseases, as seen in the cholera outbreak and resurgence of pellagra; in anticipation of this, RHIS data should be prioritized since it can facilitate rapid detection as conditions emerge and guide an informed response. Indeed, the level of granularity achieved with RHIS data is useful to understand district-level variability, as well as identify highly affected services. RHIS provides a unique opportunity to track the recovery process, particularly for indicators such as family planning or immunization for which a full recovery (not only returning to pre-shock levels but also recovering the losses accrued during periods of disruption) is a programmatic goal. Fourth, effective coordination mechanisms and strong leadership are critical in an emergency, mainly when massive solidarity exists and new stakeholders come in. We have learned that the existing tools between government agencies and partners were essential to avoid or minimize anarchy in the response and to direct aid to the most vulnerable areas during Idai.

### Limitations

This study has some notable limitations. First, districts could have experienced disruptions to service delivery after the cyclone even without significant levels of destruction. Because districts were classified by severity level based on the number of people affected, some districts could have been misclassified when estimating relative losses. Second, we relied on routine data, which may have quality issues, including missing data. Indeed, our analysis included a small number of outliers; however, these were not influential. Third, we were not able to track aid directed to each district; therefore, we missed understanding whether the recovery had any association with the resources allocated. Fourth, given the study design (uncontrolled ITSA) and lack of covariates at the district level, causal inference should be avoided and result interpretation should be conservative. Fifth, although we did not statistically test for lead and lagged effects, our data exploration (by plotting individual district time series) did not suggest such patterns. Despite the limitations, the results presented are robust and are consistent with what could have been expected after an external shock of Idai’s magnitude, with the added advantage of quantifying the effects across a set of essential service delivery indicators, using routine data that are frequently overlooked to track health system performance during and post shocks.

## CONCLUSION

This study provides evidence of the negative impacts of extreme weather events on women’s and children’s ability to access essential evidence-based interventions. It also showcases how routine data is useful for tracking health system performance and resilience during and after shocks; therefore, it should be used and prioritized to guide decision making. Overall, Cyclone Idai led to massive disruptions in health service delivery, with all elements of maternal and child health services showing meaningful and statistically significant decreases immediately following the cyclone. Recovery to pre-Idai trends occurred quickly for most indicators, although highly affected districts took relatively much longer. However, describing the specific characteristics that most influenced the health system’s recovery and accumulated losses should be investigated to more fully picture the recovery process. Finally, despite the focus on a single dimension of system resilience (ability to recover), this study contributes to evidence on features of health system resilience and alternative methods for assessing them.

## Supplementary Material

GHSP-D-21-00796-supplement.pdf

## Translation

**Em Português**

**Perdas e Recuperação dos Serviços de Saúde Materno-Infantil após o Ciclone Idai em Moçambique: Uma Análise Série Temporal Interrompida sem Controlos**

**Principais Resultados**

- Imediatamente após o Ciclone Idai, foram observadas perdas consideráveis na prestação de serviços, tendo sido maiores nos distritos severamente afectados. No geral, as primeiras consultas de pré-natais, consultas pós-parto realizadas dentro de 3 a 7 dias após o parto, novas usuárias de planeamento familiar, vacinação contra o sarampo, primeiras consultas de crianças em risco e crianças menores de 1 ano completamente imunizadas, foram os indicadores mais afectados.
- Três meses após o ciclone, observou-se uma recuperação dos indicadores para níveis iguais ou superiores ao período antes do Idai, em todos os distritos. A rápida recuperação ilustra a resiliência do Sistema de Saúde de Moçambique, particularmente na região centro.
- Métodos de pesquisa de implementação combinados com a disponibilidade de dados de rotina de alta qualidade, podem fornecer evidência relevante para apoiar a formulação de políticas e decisões estratégicas, particularmente em cenários de eventos climáticos extremos, como o Ciclone Idai. Portanto, eles devem ser priorizados.

**Principais Implicações**

Embora a prestação de cuidados de saúde materno-infantil seja impactada negativamente por eventos climáticos extremos, as lições aprendidas com o processo de recuperação podem ajudar a fortalecer os sistemas de saúde. Os formuladores de políticas devem priorizar os dados de rotina do sistema de informação de saúde, como uma ferramenta valiosa para monitorar a resiliência do sistema de saúde.

**Resumo**

**Introdução:** Eventos climáticos extremos relacionados às mudanças climáticas têm aumentado em frequência e intensidade, ameaçando a saúde das pessoas, principalmente em locais com sistemas de saúde fracos. Em Março de 2019, o ciclone Idai devastou a região centro de Moçambique, causando destruição de infraestrutura, deslocamento de população e mortes. Avaliamos o impacto do Idai nos serviços de saúde materno-infantil e a recuperação nas províncias de Sofala e Manica.

**Métodos:** Usando dados de rotina mensais de nível distrital de Novembro de 2016 a Março de 2020, realizamos uma análise de série temporal interrompida não controlada, para avaliar mudanças em 10 indicadores de saúde materno-infantil, em todos os 25 distritos, antes e depois do Idai. Aplicamos um modelo binomial negativo hierárquico bayesiano, com intercepção e inclinação aleatórias a nível de distrito, para estimar as perdas dos serviços de saúde atribuível ao Idai, bem como sua recuperação.

**Resultados:** Dos 4,44 milhões de habitantes de Sofala e Manica, 1,83 (41,2%) milhões foram afectadas. Buzi, Nhamatanda e Dondo (todos na província de Sofala), tiveram a maior proporção de pessoas afectadas. Após o Idai, todos os 10 indicadores apresentaram uma redução abrupta e considerável. As primeiras consultas pré-natais por 100.000 mulheres em idade reprodutiva, diminuíram em 23% (intervalo de confiança de 95% [IC]=, 0,62, –0,96) em Março e 11% (IC 95%=, 0,75, –1,07) em Abril. As vacinações de BCG por 1.000 crianças menores de 5 anos diminuíram em 21% (IC 95% =, 0,69, –0,90) e as vacinas contra o sarampo diminuíram em 25% (IC 95% =, 0,64, –0,87) em Março e permaneceram semelhantes em Abril. Três meses após o ciclone, quase todos os distritos recuperaram para os níveis anteriores ao Idai, incluindo Buzi, que apresentou um aumento relativo de 22% e 13% no número de primeiras consultas pré-natais e vacinas BCG, respectivamente.

**Conclusão:** Encontramos perdas consideráveis nos serviços de saúde imediatamente após o Idai, com maior impacto nos distritos mais afectados. Estes resultados sugerem uma recuperação impressionante após o Idai, enfatizando a necessidade de construir Sistemas de Saúde Resilientes para garantir cuidados de saúde de qualidade durante e após desastres naturais.

**Peer Reviewed**

## References

[1] United Nations Office for Disaster Risk Reduction (UNDRR). *Human Cost of Disasters: An Overview of the Last 20 Years 2000-2019*. UNDRR; 2020. Accessed June 24, 2022. https://www.undrr.org/media/48008/download

[2] Alderman K Turner LR Tong S. Floods and human health: a systematic review. Environ Int. 2012;47:37–47. 10.1016/j.envint.2012.06.003. 22750033

[3] Callaghan WM Rasmussen SA Jamieson DJ. Health concerns of women and infants in times of natural disasters: lessons learned from Hurricane Katrina. Matern Child Health J. 2007;11(4):307–311. 10.1007/s10995-007-0177-4. 17253147

[4] Watts N Adger WN Agnolucci P. Health and climate change: policy responses to protect public health. Lancet. 2015;386(10006):1861–1914. 10.1016/S0140-6736(15)60854-6. 26111439

[5] Brunson J. Maternal, newborn, and child health after the 2015 Nepal earthquakes: an investigation of the long-term gendered impacts of disasters. Matern Child Health J. 2017;21(12):2267–2273. 10.1007/s10995-017-2350-8. 28755049

[6] Tees MT Harville EW Xiong X Buekens P Pridjian G Elkind-Hirsch K. Hurricane Katrina-related maternal stress, maternal mental health, and early infant temperament. Matern Child Health J. 2010;14(4):511–518. 10.1007/s10995-009-0486-x. 19554438 PMC3472436

[7] Man RXG Lack DA Wyatt CE Murray V. The effect of natural disasters on cancer care: a systematic review. Lancet Oncol. 2018;19(9):e482–e499. 10.1016/S1470-2045(18)30412-1. 30191852

[8] Watson JT Gayer M Connolly MA. Epidemics after natural disasters. Emerg Infect Dis. 2007;13(1):1–5. 10.3201/eid1301.060779. 17370508 PMC2725828

[9] Fundter DQP Jonkman B Beerman S. Health impacts of large-scale floods: governmental decision-making and resilience of the citizens. Prehosp Disaster Med. 2008;23(Suppl 2):S70–S73. 10.1017/S1049023X00021282. 18935963

[10] Assanangkornchai S Tangboonngam S Edwards JG. The flooding of Hat Yai: predictors of adverse emotional responses to a natural disaster. Stress Health. 2004;20(2):81–89. 10.1002/smi.999

[11] Stephens KU Sr Grew D Chin K. Excess mortality in the aftermath of Hurricane Katrina: a preliminary report. Disaster Med Public Health Prep. 2007;1(1):15–20. 10.1097/DMP.0b013e3180691856. 18388597

[12] Parker G Lie D Siskind DJ. Mental health implications for older adults after natural disasters – a systematic review and meta-analysis. Int Psychogeriatr. 2016;28(1):11–20. 10.1017/S1041610215001210. 26212132

[13] Tong VT Zotti ME Hsia J. Impact of the Red River catastrophic flood on women giving birth in North Dakota, 1994–2000. Matern Child Health J. 2011;15(3):281–288. 10.1007/s10995-010-0576-9. 20204482

[14] Glynn LM Wadhwa PD Dunkel-Schetter C Chicz-DeMet A Sandman CA. When stress happens matters: effects of earthquake timing on stress responsivity in pregnancy. Am J Obstet Gynecol. 2001;184(4):637–642. 10.1067/mob.2001.111066. 11262465

[15] Datar A Liu J Linnemayr S Stecher C. The impact of natural disasters on child health and investments in rural India. Soc Sci Med. 2013;76(1):83–91. 10.1016/j.socscimed.2012.10.008. 23159307 PMC3544338

[16] Baten A Wallemacq P van Loenhout JAF Guha-Sapir D. Impact of recurrent floods on the utilization of maternal and newborn healthcare in Bangladesh. Matern Child Health J. 2020;24(6):748–758. 10.1007/s10995-020-02917-3. 32285334

[17] Klinger C Landeg O Murray V. Power outages, extreme events and health: a systematic review of the literature from 2011–2012. PLoS Curr. 2014;6:6. 24459613 10.1371/currents.dis.04eb1dc5e73dd1377e05a10e9edde673 PMC3879211

[18] Kruk ME Myers M Varpilah ST Dahn BT. What is a resilient health system? Lessons from Ebola. Lancet. 2015;385(9980):1910–1912. 10.1016/S0140-6736(15)60755-3. 25987159

[19] Fridell M Edwin S von Schreeb J Saulnier DD. Health system resilience: what are we talking about? A scoping review mapping characteristics and keywords. Int J Health Policy Manag. 2019;9(1):6–16. 10.15171/ijhpm.2019.71. 31902190 PMC6943300

[20] Grimm PY Merten S Wyss K. Evidence of health system resilience in Myanmar during Cyclone Nargis: a qualitative analysis. BMJ Open. 2021;11(9):e050700. 10.1136/bmjopen-2021-050700. 34551949 PMC8461277

[21] Koeva S Rohova M. Health system resilience: concept development. J IMAB 2020;26(3):3251–3258. 10.5272/jimab.2020263.3251

[22] Lequechane JD Mahumane A Chale F. Mozambique’s response to cyclone Idai: how collaboration and surveillance with water, sanitation and hygiene (WASH) interventions were used to control a cholera epidemic. Infect Dis Poverty. 2020;9(1):68. 10.1186/s40249-020-00692-5. 32546268 PMC7298796

[23] Devi S. Cyclone Idai: 1 month later, devastation persists. Lancet. 2019;393(10181):1585. 10.1016/S0140-6736(19)30892-X. 31007189

[24] Ministerio da Saude - MISAU/Moçambique; Instituto Nacional de Estatística - INE/Moçambique; ICF International. *Moçambique Inquérito Demográfico e de Saúde 2011*. MISA/Moçambique, INE/Moçambique, ICF International; 2013. Accessed June 24, 2022. https://dhsprogram.com/pubs/pdf/fr266/fr266.pdf

[25] Instituto Nacional de Estatistica (INE). *Relatório Final Do Inquérito Ao Orçamento Familiar – IOF2014/15*. INE; 2015. Accessed June 24, 2022. http://www.ine.gov.mz/operacoes-estatisticas/inqueritos/inquerito-sobre-orcamento-familiar

[26] Wagenaar BH Augusto O Beste J. The 2014–2015 Ebola virus disease outbreak and primary healthcare delivery in Liberia: Time-series analyses for 2010–2016. PLoS Med. 2018;15(2):e1002508. 10.1371/journal.pmed.1002508. 29462138 PMC5819774

[27] Xiao H Augusto O Wagenaar BH. Reflection on modern methods: a common error in the segmented regression parameterization of interrupted time-series analyses. Int J Epidemiol. 2021;50(3):1011–1015. 10.1093/ije/dyaa148. 33097937 PMC8271192

[28] Lopez Bernal J Soumerai S Gasparrini A. A methodological framework for model selection in interrupted time series studies. J Clin Epidemiol. 2018;103:82–91. 10.1016/j.jclinepi.2018.05.026. 29885427

[29] Bernal JL Cummins S Gasparrini A. Interrupted time series regression for the evaluation of public health interventions: a tutorial. Int J Epidemiol. 2017;46(1):348–355. 10.1093/ije/dyw098. 27283160 PMC5407170

[30] Araujo S Dade A Zacarias MF Chipembe CS Maunze XH Singano CC. *Final Report of the Multiple Indicator Cluster Survey 2008*. National Statistics Institute; 2009.

[31] Mozambique National Institute for Disaster Management (INGC). Mozambique — Baseline Assessment - Cyclone Idai - Round 6. INGC; 2019. Accessed June 24, 2022. https://displacement.iom.int/system/tdf/reports/Mozambique_Baseline_Assessment_Cyclone_IDAI_Round_6.pdf

[32] Demidenko E. *Mixed Models: Theory and Applications with R*. John Wiley & Sons; 2013.

[33] Bolker BM Brooks ME Clark CJ. Generalized linear mixed models: a practical guide for ecology and evolution. Trends Ecol Evol. 2009;24(3):127–135. 10.1016/j.tree.2008.10.008. 19185386

[34] Congdon PD. *Bayesian Hierarchical Models: With Applications Using R*. Chapman and Hall/CRC; 2019.

[35] The R Project for Statistical Computing. Accessed June 24, 2022. https://www.r-project.org/

[36] Bürkner PC. brms: an R package for Bayesian multilevel models using Stan. J Stat Softw. 2017;80(1). 10.18637/jss.v080.i01

[37] Mozambique National Institute of Statistics (INE). *Mozambique Population and Housing Census 2017*. INE; 2017. Accessed June 24, 2022. http://www.ine.gov.mz/iv-rgph-2017/mocambique/censo-2017-brochura-dos-resultados-definitivos-do-iv-rgph-nacional.pdf/view

[38] Lederman SA Rauh V Weiss L. The effects of the World Trade Center event on birth outcomes among term deliveries at three lower Manhattan hospitals. Environ Health Perspect. 2004;112(17):1772–1778. 10.1289/ehp.7348. 15579426 PMC1253672

[39] Goodman A. In the aftermath of disasters: the impact on women’s health. Critical Care Obstet Gynecol. 2016;2(6):29.

[40] Mozambique: Children living in storm affected areas face worsening food insecurity and nutrition crisis six months after Cyclone Idai. UNICEF. September 14, 2019. Accessed June 24, 2022. https://www.unicef.org/mozambique/en/press-releases/mozambique-children-living-storm-affected-areas-face-worsening-food-insecurity-and

[41] Rodriguez-Llanes JM Ranjan-Dash S Degomme O Mukhopadhyay A Guha-Sapir D. Child malnutrition and recurrent flooding in rural eastern India: a community-based survey. BMJ Open. 2011;1(2):e000109. 10.1136/bmjopen-2011-000109. 22080535 PMC3208901

[42] Béné C Wood RG Newsham A Davies M. Resilience: new utopia or new tyranny? Reflection about the potentials and limits of the concept of resilience in relation to vulnerability reduction programmes. IDS Work Pap. 2012;2012(405):1–61. 10.1111/j.2040-0209.2012.00405.x

[43] Biddle L Wahedi K Bozorgmehr K. Health system resilience: a literature review of empirical research. Health Policy Plan. 2020;35(8):1084–1109. 10.1093/heapol/czaa032. 32529253 PMC7553761

[44] Barasa EW Cloete K Gilson L. From bouncing back, to nurturing emergence: reframing the concept of resilience in health systems strengthening. Health Policy Plan. 2017;32(Suppl 3):iii91–iii94. 10.1093/heapol/czx118. 29149319 PMC6626547

[45] Folke C Carpenter SR Walker B Scheffer M Chapin T Rockström J. Resilience thinking: integrating resilience, adaptability and transformability. Ecol Soc. 2010;15(4):art20. 10.5751/ES-03610-150420

[46] World Health Organization (WHO). *Pillars of Strength: How Embedded Research Supports Resilient Health Systems in Mozambique: Story of Change*. WHO; 2020. Accessed July 6, 2022. https://apps.who.int/iris/handle/10665/333898

