# Supplementary material for: Maternal and Child Health Care Service Disruptions and Recovery in Mozambique After Cyclone Idai: An Uncontrolled Interrupted Time Series Analysis
Source: Glob Health Sci Pract. 2022 Sep 15;10(Suppl 1):e2100796. doi: 10.9745/GHSP-D-21-00796 (PMC9476482; doi:10.9745/GHSP-D-21-00796)
Supplement: GHSP-D-21-00796-supplement.pdf [file GHSP-D-21-00796-supplement.pdf]

Cite this article as: Fernandes Q, Augusto O, Chicumbe S, et al. Maternal and child health care service disruptions and recovery in Mozambique after Cyclone Idai: an uncontrolled interrupted time series analysis. *Glob Health Sci Pract*. 2022;10(Suppl 1):e2100796.

Supplementary materials

Negative binomial regression coefficients (in the log-scale) for each outcome

Table S1 - First antenatal care visit

Coefficient	Estimate	Est.Error	l-95% CI	u-95% CI
Intercept	-4.6732	0.0384	-4.7479	-4.598
timec	-0.0012	0.0012	-0.0035	0.0011
idai	0.0807	0.0333	0.0157	0.1454
idai.month	-0.2563	0.1101	-0.4794	-0.0407
idai.month2	-0.1121	0.0904	-0.294	0.0636
idai.post	-0.0044	0.0036	-0.0116	0.0027
as.factorindmonth1	0.0275	0.0176	-0.0062	0.0624
as.factorindmonth2	-0.0194	0.0176	-0.0541	0.0149
as.factorindmonth3	0.1221	0.0175	0.0883	0.1569
as.factorindmonth4	0.0357	0.0193	-0.0022	0.0734
as.factorindmonth5	0.0192	0.0196	-0.0193	0.0571
as.factorindmonth6	0.0629	0.0199	0.024	0.1021
as.factorindmonth7	0.0647	0.0195	0.027	0.1032
as.factorindmonth8	0.0032	0.0194	-0.034	0.0413
as.factorindmonth9	0.0457	0.0192	0.0085	0.0834
as.factorindmonth10	0.0343	0.0192	-0.0034	0.0715
as.factorindmonth11	-0.0425	0.0194	-0.0808	-0.0044
SD Random-Effects				
sd(Intercept)	0.1732	0.0304	0.125	0.2424
sd(timec)	0.0048	0.001	0.003	0.0071
sd(idai)	0.1171	0.0313	0.058	0.1821
sd(idai.month)	0.5119	0.0807	0.3774	0.6906
sd(idai.month2)	0.4085	0.0676	0.2971	0.5594
sd(idai.post)	0.012	0.0034	0.0053	0.0189
Random-effects Correlations				
cor(Intercept,timec)	-0.1824	0.2105	-0.5599	0.2453
cor(Intercept,idai)	-0.0002	0.2273	-0.4437	0.4333
cor(timec,idai)	-0.0796	0.2507	-0.5326	0.4334
cor(Intercept,idai.month)	0.0575	0.1861	-0.3069	0.4106
cor(timec,idai.month)	0.2694	0.1949	-0.1314	0.62
cor(idai,idai.month)	-0.3687	0.1965	-0.7057	0.0521
cor(Intercept,idai.month2)	-0.0033	0.1899	-0.372	0.363
cor(timec,idai.month2)	0.2198	0.2029	-0.1895	0.5914
cor(idai,idai.month2)	-0.2661	0.2126	-0.6422	0.1812
cor(idai.month,idai.month2)	0.9014	0.063	0.7389	0.9802
cor(Intercept,idai.post)	0.2251	0.2267	-0.2377	0.6364
cor(timec,idai.post)	-0.2988	0.242	-0.7192	0.213
cor(idai,idai.post)	-0.7061	0.1846	-0.9358	-0.2319
cor(idai.month,idai.post)	0.0872	0.2248	-0.3625	0.4997
cor(idai.month2,idai.post)	0.1119	0.2243	-0.3449	0.5259
shape	71.9149	3.9534	64.4101	79.8854

Cite this article as: Fernandes Q, Augusto O, Chicumbe S, et al. Maternal and child health care service disruptions and recovery in Mozambique after Cyclone Idai: an uncontrolled interrupted time series analysis. *Glob Health Sci Pract*. 2022;10(Suppl 1):e2100796.

Table S2 - At least 2 doses of intermittent preventive treatment prophylaxis (IPTp2)

Coefficient	Estimate	Est.Error	l-95% CI	u-95% CI
Intercept	-5.3721	0.0865	-5.5432	-5.2031
timec	0.005	0.0023	0.0004	0.0095
idai	0.0287	0.0503	-0.0696	0.1269
idai.month	-0.1394	0.0944	-0.3267	0.0477
idai.month2	-0.1448	0.1039	-0.3496	0.0563
idai.post	0.0029	0.006	-0.0088	0.0149
as.factorindmonth1	-0.0171	0.0351	-0.0857	0.0507
as.factorindmonth2	-0.0273	0.035	-0.0954	0.0397
as.factorindmonth3	0.0062	0.0354	-0.0625	0.0763
as.factorindmonth4	-0.0662	0.0389	-0.1418	0.0103
as.factorindmonth5	-0.0231	0.0388	-0.0988	0.0541
as.factorindmonth6	-0.0599	0.0389	-0.1357	0.0158
as.factorindmonth7	-0.0484	0.0387	-0.1237	0.0283
as.factorindmonth8	0.0453	0.0386	-0.0297	0.1214
as.factorindmonth9	-0.0347	0.0385	-0.1095	0.0412
as.factorindmonth10	-0.0569	0.0385	-0.1329	0.0186
as.factorindmonth11	0.0015	0.0385	-0.0745	0.0772
SD Random-Effects				
sd(Intercept)	0.3925	0.0647	0.2876	0.5412
sd(timec)	0.0098	0.0019	0.0066	0.0139
sd(idai)	0.1088	0.0506	0.0123	0.2121
sd(idai.month)	0.2695	0.1056	0.0553	0.4812
sd(idai.month2)	0.351	0.1052	0.1408	0.563
sd(idai.post)	0.0132	0.0058	0.0019	0.0249
Random-effects Correlations				
cor(Intercept,timec)	-0.3157	0.1839	-0.6376	0.0728
cor(Intercept,idai)	-0.0079	0.2973	-0.5827	0.5643
cor(timec,idai)	-0.2701	0.3032	-0.7605	0.4002
cor(Intercept,idai.month)	0.0025	0.2564	-0.4893	0.4881
cor(timec,idai.month)	0.0092	0.2643	-0.4869	0.5315
cor(idai,idai.month)	0.3394	0.3095	-0.3441	0.8343
cor(Intercept,idai.month2)	0.1312	0.2302	-0.3212	0.5636
cor(timec,idai.month2)	-0.0777	0.2404	-0.5313	0.3961
cor(idai,idai.month2)	0.4135	0.2934	-0.2607	0.8594
cor(idai.month,idai.month2)	0.6379	0.2435	0.0004	0.9373
cor(Intercept,idai.post)	0.1413	0.2824	-0.4373	0.6612
cor(timec,idai.post)	-0.5827	0.2506	-0.9143	0.0347
cor(idai,idai.post)	-0.0753	0.3481	-0.6875	0.6322
cor(idai.month,idai.post)	0.0041	0.3215	-0.6429	0.594
cor(idai.month2,idai.post)	0.059	0.3123	-0.5851	0.6215
shape	17.3182	0.8914	15.6191	19.0981

Cite this article as: Fernandes Q, Augusto O, Chicumbe S, et al. Maternal and child health care service disruptions and recovery in Mozambique after Cyclone Idai: an uncontrolled interrupted time series analysis. *Glob Health Sci Pract*. 2022;10(Suppl 1):e2100796.

Table S3 - Institutional delivery

Coefficient	Estimate	Est.Error	l-95% CI	u-95% CI
Intercept	-5.1143	0.039	-5.1913	-5.038
timec	0.0047	0.0011	0.0024	0.0069
idai	0.0004	0.0232	-0.045	0.0465
idai.month	-0.1414	0.0403	-0.2201	-0.0634
idai.month2	-0.1025	0.0371	-0.1745	-0.0292
idai.post	-0.0117	0.0024	-0.0165	-0.0069
as.factorindmonth1	-0.0606	0.0132	-0.0864	-0.0345
as.factorindmonth2	-0.0405	0.0133	-0.0673	-0.0151
as.factorindmonth3	-0.0116	0.0133	-0.0378	0.0144
as.factorindmonth4	0.0171	0.0145	-0.0117	0.0455
as.factorindmonth5	0.0783	0.0147	0.0497	0.1069
as.factorindmonth6	0.0436	0.0147	0.0148	0.0724
as.factorindmonth7	0.0453	0.0146	0.0164	0.0738
as.factorindmonth8	-0.0024	0.0145	-0.0314	0.0265
as.factorindmonth9	0.034	0.0146	0.0053	0.0623
as.factorindmonth10	0.1374	0.0141	0.1095	0.1651
as.factorindmonth11	0.0639	0.0144	0.036	0.0924
SD Random-Effects				
sd(Intercept)	0.1825	0.03	0.1355	0.251
sd(timec)	0.005	0.0009	0.0036	0.007
sd(idai)	0.0775	0.0224	0.0378	0.1255
sd(idai.month)	0.1402	0.0347	0.0791	0.2149
sd(idai.month2)	0.1231	0.0328	0.0637	0.1935
sd(idai.post)	0.0068	0.0024	0.0023	0.0119
Random-effects Correlations				
cor(Intercept,timec)	-0.3024	0.1844	-0.6265	0.0795
cor(Intercept,idai)	0.0149	0.2401	-0.4511	0.4734
cor(timec,idai)	-0.2314	0.2374	-0.6443	0.2746
cor(Intercept,idai.month)	0.0573	0.2204	-0.3779	0.4832
cor(timec,idai.month)	0.3176	0.2144	-0.1305	0.6924
cor(idai,idai.month)	0.1734	0.2715	-0.3536	0.6881
cor(Intercept,idai.month2)	-0.032	0.2295	-0.4718	0.4194
cor(timec,idai.month2)	0.2753	0.2275	-0.1951	0.6777
cor(idai,idai.month2)	0.2558	0.2716	-0.3041	0.7446
cor(idai.month,idai.month2)	0.7708	0.1533	0.3863	0.9622
cor(Intercept,idai.post)	0.1902	0.2563	-0.33	0.6507
cor(timec,idai.post)	-0.4439	0.2349	-0.82	0.0838
cor(idai,idai.post)	0.0193	0.305	-0.5484	0.632
cor(idai.month,idai.post)	-0.3612	0.2682	-0.8168	0.207
cor(idai.month2,idai.post)	-0.3255	0.2763	-0.7983	0.2418
shape	154.4749	9.3495	136.8563	173.7925

Cite this article as: Fernandes Q, Augusto O, Chicumbe S, et al. Maternal and child health care service disruptions and recovery in Mozambique after Cyclone Idai: an uncontrolled interrupted time series analysis. *Glob Health Sci Pract*. 2022;10(Suppl 1):e2100796.

Table S4 - New Family Planning users

Coefficient	Estimate	Est.Error	l-95% CI	u-95% CI
Intercept	-3.6767	0.0942	-3.86	-3.49
timec	0.0131	0.0037	0.0058	0.0205
idai	0.2954	0.0897	0.1174	0.472
idai.month	-0.4454	0.1454	-0.7284	-0.1584
idai.month2	-0.3687	0.1404	-0.6424	-0.0881
idai.post	-0.0938	0.0122	-0.118	-0.0699
as.factorindmonth1	-0.5924	0.0665	-0.7228	-0.4629
as.factorindmonth2	-0.739	0.0667	-0.872	-0.61
as.factorindmonth3	-0.6373	0.0667	-0.7669	-0.5067
as.factorindmonth4	-0.5788	0.0724	-0.7193	-0.439
as.factorindmonth5	-0.6143	0.0736	-0.7559	-0.4681
as.factorindmonth6	-0.334	0.078	-0.4879	-0.1804
as.factorindmonth7	-0.5785	0.0727	-0.7199	-0.4353
as.factorindmonth8	-0.6422	0.0724	-0.7832	-0.4986
as.factorindmonth9	-0.6315	0.072	-0.7703	-0.4918
as.factorindmonth10	-0.15	0.0726	-0.2914	-0.0079
as.factorindmonth11	-0.0031	0.0719	-0.1419	0.1405
SD Random-Effects				
sd(Intercept)	0.3795	0.0659	0.2686	0.5267
sd(timec)	0.0145	0.0031	0.0091	0.0211
sd(idai)	0.1077	0.0785	0.0041	0.2892
sd(idai.month)	0.158	0.1125	0.0068	0.4212
sd(idai.month2)	0.1293	0.0983	0.0053	0.3662
sd(idai.post)	0.0348	0.01	0.0168	0.0558
Random-effects Correlations				
cor(Intercept,timec)	-0.7535	0.118	-0.9169	-0.4643
cor(Intercept,idai)	-0.0707	0.3452	-0.7073	0.6128
cor(timec,idai)	0.0015	0.3494	-0.6638	0.6715
cor(Intercept,idai.month)	-0.0018	0.355	-0.6753	0.6673
cor(timec,idai.month)	0.1579	0.3601	-0.5806	0.7755
cor(idai,idai.month)	-0.0542	0.377	-0.7463	0.6743
cor(Intercept,idai.month2)	-0.0553	0.3619	-0.7207	0.653
cor(timec,idai.month2)	0.1109	0.3702	-0.634	0.7565
cor(idai,idai.month2)	-0.03	0.3772	-0.7211	0.6893
cor(idai.month,idai.month2)	0.0727	0.3845	-0.6701	0.7629
cor(Intercept,idai.post)	0.0421	0.2409	-0.4377	0.4933
cor(timec,idai.post)	0.067	0.2677	-0.4435	0.5987
cor(idai,idai.post)	-0.1917	0.3705	-0.7962	0.5904
cor(idai.month,idai.post)	0.0675	0.3621	-0.6468	0.7182
cor(idai.month2,idai.post)	0.1491	0.3702	-0.597	0.785
shape	4.6545	0.2083	4.2573	5.074

Cite this article as: Fernandes Q, Augusto O, Chicumbe S, et al. Maternal and child health care service disruptions and recovery in Mozambique after Cyclone Idai: an uncontrolled interrupted time series analysis. *Glob Health Sci Pract*. 2022;10(Suppl 1):e2100796.

Table S5 - Number of Measles doses vaccination

Coefficient	Estimate	Est.Error	l-95% CI	u-95% CI
Intercept	-4.871	0.038	-4.9453	-4.7965
timec	0.0035	0.0013	0.001	0.0061
idai	0.1434	0.0443	0.0569	0.2311
idai.month	-0.287	0.0773	-0.4421	-0.1344
idai.month2	-0.2756	0.0954	-0.4664	-0.0896
idai.post	-0.0244	0.0054	-0.0348	-0.0138
as.factorindmonth1	-0.2088	0.0327	-0.2727	-0.1441
as.factorindmonth2	-0.2908	0.033	-0.356	-0.2271
as.factorindmonth3	-0.1903	0.0335	-0.2562	-0.1258
as.factorindmonth4	-0.163	0.0363	-0.234	-0.0918
as.factorindmonth5	-0.2368	0.0365	-0.3098	-0.1653
as.factorindmonth6	-0.1866	0.037	-0.2584	-0.1131
as.factorindmonth7	-0.2949	0.0364	-0.3665	-0.2238
as.factorindmonth8	-0.1466	0.0363	-0.2172	-0.0749
as.factorindmonth9	-0.1677	0.0361	-0.2391	-0.0963
as.factorindmonth10	-0.0337	0.0359	-0.1044	0.0356
as.factorindmonth11	-0.1316	0.0361	-0.2019	-0.0611
SD Random-Effects				
sd(Intercept)	0.1301	0.0264	0.0867	0.1901
sd(timec)	0.0032	0.0014	0.0004	0.006
sd(idai)	0.0581	0.0412	0.0024	0.1519
sd(idai.month)	0.1397	0.0766	0.0092	0.2985
sd(idai.month2)	0.3106	0.1091	0.0843	0.5286
sd(idai.post)	0.0101	0.0056	0.0007	0.0218
Random-effects Correlations				
cor(Intercept,timec)	-0.2838	0.2923	-0.7393	0.3869
cor(Intercept,idai)	-0.0526	0.3427	-0.6943	0.6232
cor(timec,idai)	0.0866	0.3641	-0.6211	0.7487
cor(Intercept,idai.month)	0.0899	0.3143	-0.55	0.6661
cor(timec,idai.month)	0.1804	0.3431	-0.5409	0.7714
cor(idai,idai.month)	-0.0076	0.3686	-0.684	0.6926
cor(Intercept,idai.month2)	0.0308	0.2501	-0.4492	0.5138
cor(timec,idai.month2)	0.3162	0.2966	-0.3283	0.8055
cor(idai,idai.month2)	0.1106	0.3513	-0.588	0.7444
cor(idai.month,idai.month2)	0.4918	0.3225	-0.3172	0.9113
cor(Intercept,idai.post)	0.072	0.3113	-0.5587	0.6435
cor(timec,idai.post)	-0.2725	0.349	-0.8144	0.5037
cor(idai,idai.post)	-0.3233	0.3917	-0.8842	0.5419
cor(idai.month,idai.post)	0.0023	0.3587	-0.6891	0.6658
cor(idai.month2,idai.post)	-0.2012	0.3183	-0.7571	0.4633
shape	19.1058	0.9504	17.2945	21.0257

Cite this article as: Fernandes Q, Augusto O, Chicumbe S, et al. Maternal and child health care service disruptions and recovery in Mozambique after Cyclone Idai: an uncontrolled interrupted time series analysis. *Glob Health Sci Pract*. 2022;10(Suppl 1):e2100796.

Table S6 - Number of BCG doses vaccination

Coefficient	Estimate	Est.Error	l-95% CI	u-95% CI
Intercept	-4.7885	0.0369	-4.8629	-4.7153
timec	0.0006	0.0011	-0.0015	0.0029
idai	0.124	0.0351	0.0557	0.1933
idai.month	-0.2401	0.0674	-0.3725	-0.1091
idai.month2	-0.2327	0.1013	-0.4326	-0.0306
idai.post	-0.0164	0.0044	-0.0251	-0.0077
as.factorindmonth1	-0.1688	0.0267	-0.2217	-0.1169
as.factorindmonth2	-0.2275	0.0268	-0.2804	-0.1746
as.factorindmonth3	-0.1716	0.027	-0.2238	-0.1183
as.factorindmonth4	-0.1625	0.0297	-0.2202	-0.1043
as.factorindmonth5	-0.1093	0.0296	-0.1679	-0.0523
as.factorindmonth6	-0.0944	0.0299	-0.1535	-0.0367
as.factorindmonth7	-0.0761	0.0292	-0.1342	-0.0185
as.factorindmonth8	-0.1372	0.0293	-0.1949	-0.0797
as.factorindmonth9	-0.0867	0.0294	-0.1441	-0.0291
as.factorindmonth10	0.0145	0.0291	-0.0418	0.072
as.factorindmonth11	-0.0174	0.0288	-0.0749	0.0386
SD Random-Effects				
sd(Intercept)	0.1461	0.0272	0.1014	0.2083
sd(timec)	0.0031	0.001	0.001	0.0052
sd(idai)	0.0337	0.026	0.0013	0.0958
sd(idai.month)	0.1694	0.0605	0.0495	0.2919
sd(idai.month2)	0.4152	0.0829	0.2719	0.5965
sd(idai.post)	0.0088	0.0039	0.0013	0.0167
Random-effects Correlations				
cor(Intercept,timec)	-0.1481	0.2649	-0.6101	0.4134
cor(Intercept,idai)	-0.0711	0.3631	-0.7231	0.6397
cor(timec,idai)	-0.0387	0.3688	-0.7123	0.6709
cor(Intercept,idai.month)	0.0014	0.269	-0.5243	0.5149
cor(timec,idai.month)	0.4207	0.2778	-0.2102	0.8583
cor(idai,idai.month)	-0.0817	0.3724	-0.7358	0.6524
cor(Intercept,idai.month2)	0.1055	0.2094	-0.3095	0.4989
cor(timec,idai.month2)	0.4855	0.2411	-0.0611	0.8688
cor(idai,idai.month2)	-0.0236	0.3674	-0.7064	0.6762
cor(idai.month,idai.month2)	0.6597	0.2108	0.1435	0.9345
cor(Intercept,idai.post)	-0.0401	0.2872	-0.5981	0.5089
cor(timec,idai.post)	-0.1832	0.3247	-0.7245	0.5234
cor(idai,idai.post)	-0.1466	0.3855	-0.8013	0.6372
cor(idai.month,idai.post)	-0.1231	0.3248	-0.7248	0.5178
cor(idai.month2,idai.post)	-0.3487	0.2813	-0.8157	0.2686
shape	30.1834	1.4866	27.3459	33.196

Cite this article as: Fernandes Q, Augusto O, Chicumbe S, et al. Maternal and child health care service disruptions and recovery in Mozambique after Cyclone Idai: an uncontrolled interrupted time series analysis. *Glob Health Sci Pract*. 2022;10(Suppl 1):e2100796.

Table S7 - Number of DPT-Hib 3 doses vaccination

Coefficient	Estimate	Est.Error	l-95% CI	u-95% CI
Intercept	-3.8532	0.0531	-3.9582	-3.749
timec	0.0025	0.0011	0.0003	0.0047
idai	0.0898	0.0346	0.022	0.158
idai.month	-0.1839	0.065	-0.312	-0.0539
idai.month2	-0.2665	0.1096	-0.484	-0.0474
idai.post	-0.0146	0.0044	-0.0233	-0.006
as.factorindmonth1	-0.1841	0.0254	-0.2333	-0.1335
as.factorindmonth2	-0.2228	0.0253	-0.2726	-0.1736
as.factorindmonth3	-0.1497	0.0253	-0.1992	-0.1004
as.factorindmonth4	-0.1497	0.0276	-0.2044	-0.0956
as.factorindmonth5	-0.1983	0.028	-0.2536	-0.1445
as.factorindmonth6	-0.1748	0.0283	-0.2297	-0.1193
as.factorindmonth7	-0.1772	0.0276	-0.231	-0.124
as.factorindmonth8	-0.1388	0.0278	-0.1935	-0.0839
as.factorindmonth9	-0.1199	0.0274	-0.1731	-0.0657
as.factorindmonth10	-0.0157	0.0271	-0.0684	0.0372
as.factorindmonth11	-0.0756	0.0273	-0.129	-0.0229
SD Random-Effects				
sd(Intercept)	0.2409	0.0387	0.1778	0.3299
sd(timec)	0.0034	0.0011	0.0012	0.0057
sd(idai)	0.0545	0.0341	0.0028	0.1271
sd(idai.month)	0.1803	0.0637	0.0574	0.3107
sd(idai.month2)	0.469	0.0886	0.3171	0.662
sd(idai.post)	0.0113	0.0042	0.0029	0.0199
Random-effects Correlations				
cor(Intercept,timec)	-0.5195	0.2176	-0.8485	-0.0136
cor(Intercept,idai)	-0.2795	0.3298	-0.8047	0.4507
cor(timec,idai)	0.1796	0.3461	-0.539	0.7827
cor(Intercept,idai.month)	-0.1008	0.2533	-0.5768	0.4036
cor(timec,idai.month)	0.2482	0.2964	-0.3601	0.7677
cor(idai,idai.month)	0.0247	0.3543	-0.6373	0.6933
cor(Intercept,idai.month2)	-0.1388	0.1961	-0.5024	0.2541
cor(timec,idai.month2)	0.4485	0.2373	-0.0831	0.834
cor(idai,idai.month2)	0.2217	0.3389	-0.5085	0.7984
cor(idai.month,idai.month2)	0.6102	0.2118	0.1006	0.9131
cor(Intercept,idai.post)	0.0346	0.2618	-0.4867	0.5477
cor(timec,idai.post)	-0.3057	0.2943	-0.7818	0.361
cor(idai,idai.post)	-0.2773	0.3608	-0.8343	0.5251
cor(idai.month,idai.post)	-0.1268	0.3146	-0.7258	0.4799
cor(idai.month2,idai.post)	-0.3645	0.2539	-0.7995	0.1819
shape	33.9463	1.7098	30.6874	37.3343

Cite this article as: Fernandes Q, Augusto O, Chicumbe S, et al. Maternal and child health care service disruptions and recovery in Mozambique after Cyclone Idai: an uncontrolled interrupted time series analysis. *Glob Health Sci Pract*. 2022;10(Suppl 1):e2100796.

Table S8 - Fully immunized children underage of 1 year

Coefficient	Estimate	Est.Error	l-95% CI	u-95% CI
Intercept	-3.9495	0.0566	-4.0616	-3.8386
timec	0.0057	0.0014	0.0029	0.0085
idai	0.1224	0.0475	0.0312	0.2166
idai.month	-0.2749	0.0826	-0.437	-0.1115
idai.month2	-0.2341	0.0908	-0.416	-0.058
idai.post	-0.0252	0.0057	-0.0366	-0.0139
as.factorindmonth1	-0.2155	0.0369	-0.2861	-0.1417
as.factorindmonth2	-0.3152	0.0373	-0.3889	-0.2424
as.factorindmonth3	-0.1155	0.0372	-0.1887	-0.0429
as.factorindmonth4	-0.1662	0.0408	-0.245	-0.0868
as.factorindmonth5	-0.2479	0.0408	-0.3282	-0.1672
as.factorindmonth6	-0.2457	0.0414	-0.3275	-0.164
as.factorindmonth7	-0.3187	0.0415	-0.3999	-0.2368
as.factorindmonth8	-0.1874	0.0408	-0.268	-0.1079
as.factorindmonth9	-0.2012	0.0403	-0.2806	-0.1213
as.factorindmonth10	-0.0546	0.0403	-0.1334	0.024
as.factorindmonth11	-0.1499	0.0396	-0.2276	-0.0715
SD Random-Effects				
sd(Intercept)	0.235	0.0392	0.1712	0.3224
sd(timec)	0.0033	0.0015	0.0005	0.0062
sd(idai)	0.0377	0.0289	0.0014	0.1079
sd(idai.month)	0.1157	0.0783	0.005	0.2904
sd(idai.month2)	0.2072	0.1305	0.0099	0.4777
sd(idai.post)	0.0081	0.0049	0.0005	0.0186
Random-effects Correlations				
cor(Intercept,timec)	-0.4691	0.2646	-0.8523	0.1754
cor(Intercept,idai)	-0.0659	0.3682	-0.7352	0.6455
cor(timec,idai)	-0.0482	0.3801	-0.7355	0.6805
cor(Intercept,idai.month)	-0.0721	0.3309	-0.6854	0.5783
cor(timec,idai.month)	0.1698	0.3576	-0.5656	0.7788
cor(idai,idai.month)	-0.0116	0.3749	-0.7055	0.6937
cor(Intercept,idai.month2)	-0.0865	0.2895	-0.6406	0.5155
cor(timec,idai.month2)	0.2262	0.3411	-0.5173	0.7882
cor(idai,idai.month2)	0.0269	0.3759	-0.6879	0.7176
cor(idai.month,idai.month2)	0.3014	0.3806	-0.5528	0.8713
cor(Intercept,idai.post)	0.1912	0.3201	-0.4837	0.7386
cor(timec,idai.post)	-0.2576	0.3628	-0.8258	0.537
cor(idai,idai.post)	-0.1302	0.3831	-0.7867	0.6369
cor(idai.month,idai.post)	-0.075	0.3659	-0.7398	0.642
cor(idai.month2,idai.post)	-0.1522	0.3559	-0.7657	0.588
shape	15.427	0.7589	13.9856	16.9676

Cite this article as: Fernandes Q, Augusto O, Chicumbe S, et al. Maternal and child health care service disruptions and recovery in Mozambique after Cyclone Idai: an uncontrolled interrupted time series analysis. *Glob Health Sci Pract*. 2022;10(Suppl 1):e2100796.

Table S9 - First at-risk children's consultation

Coefficient	Estimate	Est.Error	l-95% CI	u-95% CI
Intercept	-7.0332	0.1153	-7.2626	-6.8094
timec	0.0022	0.0031	-0.004	0.0084
idai	0.2368	0.0595	0.1209	0.3545
idai.month	-0.29	0.0897	-0.4669	-0.1144
idai.month2	-0.3148	0.1341	-0.5941	-0.0592
idai.post	0.0056	0.0079	-0.0102	0.021
as.factorindmonth1	-0.1	0.0408	-0.1792	-0.0211
as.factorindmonth2	-0.166	0.041	-0.2466	-0.0865
as.factorindmonth3	0.0651	0.041	-0.0146	0.1454
as.factorindmonth4	-0.0062	0.0443	-0.0926	0.0802
as.factorindmonth5	0.0177	0.0446	-0.07	0.1038
as.factorindmonth6	-0.0091	0.0452	-0.0963	0.0805
as.factorindmonth7	0.0463	0.0451	-0.0413	0.1351
as.factorindmonth8	0.0183	0.0445	-0.0686	0.1056
as.factorindmonth9	-0.0314	0.0442	-0.1177	0.0542
as.factorindmonth10	0.0114	0.0443	-0.0747	0.0985
as.factorindmonth11	-0.0629	0.044	-0.1503	0.0243
SD Random-Effects				
sd(Intercept)	0.546	0.089	0.4023	0.7482
sd(timec)	0.0137	0.0027	0.009	0.0197
sd(idai)	0.1294	0.0606	0.0145	0.2539
sd(idai.month)	0.0897	0.0693	0.0036	0.2565
sd(idai.month2)	0.4704	0.209	0.0428	0.8663
sd(idai.post)	0.0241	0.0065	0.0121	0.038
Random-effects Correlations				
cor(Intercept,timec)	-0.3281	0.1873	-0.6531	0.0704
cor(Intercept,idai)	-0.3592	0.2798	-0.8148	0.2581
cor(timec,idai)	0.2425	0.2943	-0.3685	0.756
cor(Intercept,idai.month)	-0.0998	0.3585	-0.7333	0.6092
cor(timec,idai.month)	0.001	0.361	-0.6815	0.6897
cor(idai,idai.month)	-0.0592	0.3784	-0.7399	0.6739
cor(Intercept,idai.month2)	-0.0439	0.2353	-0.4896	0.4322
cor(timec,idai.month2)	0.1597	0.2442	-0.3454	0.6122
cor(idai,idai.month2)	-0.0696	0.3203	-0.6426	0.585
cor(idai.month,idai.month2)	0.0776	0.3858	-0.6528	0.7674
cor(Intercept,idai.post)	-0.1725	0.2352	-0.6131	0.2983
cor(timec,idai.post)	-0.5342	0.2042	-0.8518	-0.0669
cor(idai,idai.post)	0.0084	0.317	-0.5884	0.6301
cor(idai.month,idai.post)	0.1499	0.3712	-0.6103	0.7787
cor(idai.month2,idai.post)	-0.0437	0.279	-0.596	0.4848
shape	15.6737	1.0122	13.778	17.7428

Cite this article as: Fernandes Q, Augusto O, Chicumbe S, et al. Maternal and child health care service disruptions and recovery in Mozambique after Cyclone Idai: an uncontrolled interrupted time series analysis. *Glob Health Sci Pract*. 2022;10(Suppl 1):e2100796.

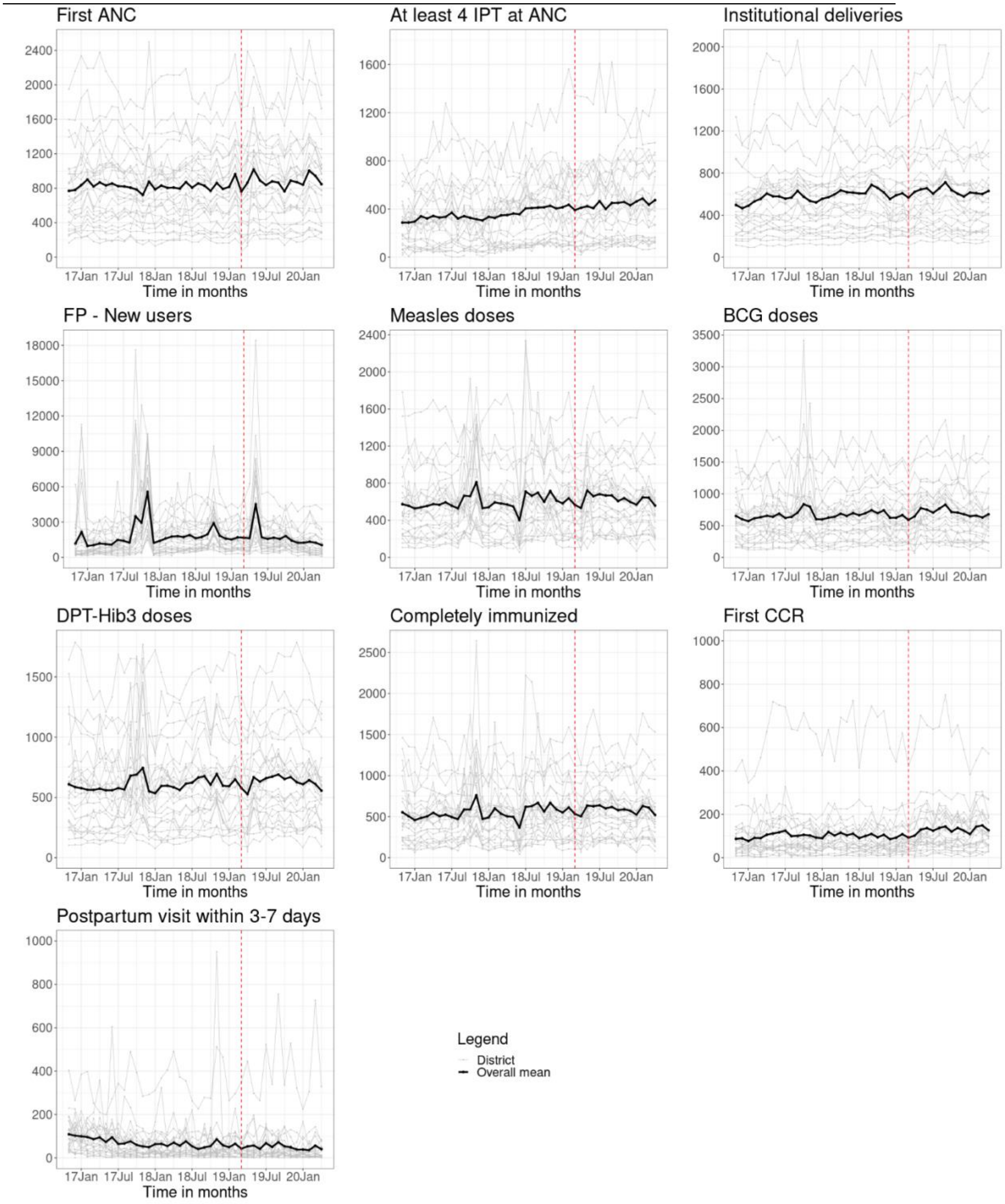

Figure S1 – Raw counts of service provision. The gray lines are for each of the 25 districts and the black solid line is for the overall mean.(1)

Cite this article as: Fernandes Q, Augusto O, Chicumbe S, et al. Maternal and child health care service disruptions and recovery in Mozambique after Cyclone Idai: an uncontrolled interrupted time series analysis. *Glob Health Sci Pract*. 2022;10(Suppl 1):e2100796.

Cite this article as: Fernandes Q, Augusto O, Chicumbe S, et al. Maternal and child health care service disruptions and recovery in Mozambique after Cyclone Idai: an uncontrolled interrupted time series analysis. *Glob Health Sci Pract*. 2022;10(Suppl 1):e2100796.

Descriptive of service count before, during and after Idai cyclone

Table T1 - First antenatal care visit (mean and standard deviation)

District/Province	2017 and 2018	March 2019	April 2019	Post
Overall	823 (453.70)	761 (450.96)	865 (549.25)	888 (479.70)
MANICA	849 (414.80)	882 (451.73)	929 (486.54)	937 (461.43)
BARUE	1031 (168.38)	1283 (NA)	1217 (NA)	1223 (53.88)
CIDADE DE CHIMOIO	1636 (227.69)	1727 (NA)	1754 (NA)	1842 (187.57)
GONDOLA	1029 (75.83)	879 (NA)	1169 (NA)	1063 (105.80)
GURO	534 (48.61)	581 (NA)	496 (NA)	557 (54.74)
MACATE	571 (176.46)	603 (NA)	669 (NA)	578 (73.53)
MACHAZE	625 (69.27)	644 (NA)	724 (NA)	747 (56.77)
MACOSSA	242 (97.82)	265 (NA)	265 (NA)	288 (32.97)
MANICA	1086 (80.74)	1302 (NA)	1287 (NA)	1304 (85.20)
MOSSURIZE	1340 (131.43)	1263 (NA)	1465 (NA)	1316 (121.66)
SUSSUNDENGA	995 (95.45)	1037 (NA)	1110 (NA)	1112 (222.85)
TAMBARA	323 (92.16)	250 (NA)	163 (NA)	266 (38.51)
VANDUZI	753 (99.40)	744 (NA)	830 (NA)	946 (83.00)
SOFALA	800 (486.34)	650 (437.90)	806 (615.19)	843 (493.12)
BUZI	896 (104.11)	66 (NA)	133 (NA)	951 (103.97)
CAIA	894 (54.27)	834 (NA)	976 (NA)	983 (106.83)
CHEMBA	433 (57.68)	426 (NA)	409 (NA)	441 (67.68)
CHERINGOMA	362 (32.64)	410 (NA)	369 (NA)	381 (31.35)
CHIBABAVA	700 (67.25)	632 (NA)	754 (NA)	739 (69.60)
CIDADE DA BEIRA	2004 (193.51)	1649 (NA)	2387 (NA)	2035 (263.73)
DONDO	921 (106.52)	728 (NA)	1137 (NA)	948 (90.78)
GORONGOSA	1062 (155.66)	1003 (NA)	1179 (NA)	1134 (136.44)
MACHANGA	296 (14.56)	227 (NA)	259 (NA)	357 (210.83)
MARINGUE	522 (76.83)	504 (NA)	541 (NA)	619 (94.23)
MARROMEU	744 (106.30)	700 (NA)	801 (NA)	749 (109.89)
MUANZA	187 (28.11)	141 (NA)	224 (NA)	215 (44.18)
NHAMATANDA	1383 (103.06)	1127 (NA)	1308 (NA)	1402 (161.54)

Cite this article as: Fernandes Q, Augusto O, Chicumbe S, et al. Maternal and child health care service disruptions and recovery in Mozambique after Cyclone Idai: an uncontrolled interrupted time series analysis. *Glob Health Sci Pract*. 2022;10(Suppl 1):e2100796.

Table T2 - At least 2 doses of intermittent preventive treatment prophylaxis (IPTp2)

District/Province	2017 and 2018	March 2019	April 2019	Post
Overall	354 (255.20)	391 (308.14)	411 (318.68)	446 (310.25)
MANICA	395 (243.99)	438 (256.64)	477 (268.45)	496 (283.00)
BARUE	386 (143.40)	711 (NA)	683 (NA)	759 (79.52)
CIDADE DE CHIMOIO	841 (174.79)	725 (NA)	729 (NA)	919 (147.25)
GONDOLA	482 (79.40)	542 (NA)	636 (NA)	715 (61.49)
GURO	362 (106.90)	430 (NA)	416 (NA)	420 (55.80)
MACATE	369 (72.34)	274 (NA)	358 (NA)	311 (58.49)
MACHAZE	121 (50.44)	153 (NA)	219 (NA)	164 (15.09)
MACOSSA	89 (31.22)	21 (NA)	19 (NA)	116 (25.39)
MANICA	614 (101.34)	660 (NA)	782 (NA)	766 (139.00)
MOSSURIZE	647 (131.54)	656 (NA)	716 (NA)	636 (115.05)
SUSSUNDENGA	310 (82.30)	318 (NA)	439 (NA)	437 (97.81)
TAMBARA	76 (28.75)	102 (NA)	55 (NA)	87 (19.01)
VANDUZI	418 (102.49)	663 (NA)	671 (NA)	626 (92.65)
SOFALA	316 (259.81)	348 (353.94)	349 (358.59)	399 (327.41)
BUZI	356 (61.28)	57 (NA)	45 (NA)	353 (75.09)
CAIA	369 (87.60)	527 (NA)	528 (NA)	500 (85.62)
CHEMBA	68 (44.07)	84 (NA)	92 (NA)	115 (20.04)
CHERINGOMA	72 (28.71)	89 (NA)	97 (NA)	121 (16.06)
CHIBABAVA	216 (37.93)	226 (NA)	192 (NA)	288 (38.24)
CIDADE DA BEIRA	949 (154.35)	1346 (NA)	1334 (NA)	1259 (201.60)
DONDO	380 (95.02)	327 (NA)	326 (NA)	457 (117.57)
GORONGOSA	451 (112.10)	412 (NA)	521 (NA)	537 (97.82)
MACHANGA	136 (28.91)	166 (NA)	208 (NA)	161 (30.50)
MARINGUE	117 (23.96)	123 (NA)	140 (NA)	145 (30.00)
MARROMEU	295 (132.01)	435 (NA)	345 (NA)	376 (59.93)
MUANZA	71 (29.63)	96 (NA)	40 (NA)	95 (29.66)
NHAMATANDA	621 (93.21)	630 (NA)	673 (NA)	777 (70.96)

Cite this article as: Fernandes Q, Augusto O, Chicumbe S, et al. Maternal and child health care service disruptions and recovery in Mozambique after Cyclone Idai: an uncontrolled interrupted time series analysis. *Glob Health Sci Pract*. 2022;10(Suppl 1):e2100796.

Table T3 - Institutional delivery

District/Province	2017 and 2018	March 2019	April 2019	Post
Overall	585 (362.05)	567 (356.71)	621 (398.04)	629 (391.47)
MANICA	602 (315.70)	643 (352.39)	684 (377.78)	674 (374.87)
BARUE	778 (151.28)	940 (NA)	1004 (NA)	962 (49.88)
CIDADE DE CHIMOIO	1313 (88.82)	1353 (NA)	1485 (NA)	1504 (86.46)
GONDOLA	701 (57.34)	649 (NA)	671 (NA)	708 (37.54)
GURO	379 (45.63)	406 (NA)	388 (NA)	431 (44.82)
MACATE	388 (44.81)	389 (NA)	365 (NA)	335 (32.46)
MACHAZE	462 (62.69)	400 (NA)	494 (NA)	476 (49.45)
MACOSSA	177 (16.47)	188 (NA)	216 (NA)	227 (18.27)
MANICA	732 (83.67)	850 (NA)	928 (NA)	926 (41.25)
MOSSURIZE	910 (140.33)	1007 (NA)	1049 (NA)	1054 (122.72)
SUSSUNDENGA	623 (54.09)	727 (NA)	731 (NA)	711 (32.77)
TAMBARA	199 (25.21)	192 (NA)	258 (NA)	189 (23.98)
VANDUZI	562 (66.93)	611 (NA)	615 (NA)	568 (26.78)
SOFALA	602 (315.70)	643 (352.39)	684 (377.78)	674 (374.87)
BUZI	778 (151.28)	940 (NA)	1004 (NA)	962 (49.88)
CAIA	1313 (88.82)	1353 (NA)	1485 (NA)	1504 (86.46)
CHEMBA	701 (57.34)	649 (NA)	671 (NA)	708 (37.54)
CHERINGOMA	379 (45.63)	406 (NA)	388 (NA)	431 (44.82)
CHIBABAVA	388 (44.81)	389 (NA)	365 (NA)	335 (32.46)
CIDADE DA BEIRA	462 (62.69)	400 (NA)	494 (NA)	476 (49.45)
DONDO	177 (16.47)	188 (NA)	216 (NA)	227 (18.27)
GORONGOSA	732 (83.67)	850 (NA)	928 (NA)	926 (41.25)
MACHANGA	910 (140.33)	1007 (NA)	1049 (NA)	1054 (122.72)
MARINGUE	623 (54.09)	727 (NA)	731 (NA)	711 (32.77)
MARROMEU	199 (25.21)	192 (NA)	258 (NA)	189 (23.98)
MUANZA	562 (66.93)	611 (NA)	615 (NA)	568 (26.78)
NHAMATANDA	1040 (92.77)	823 (NA)	911 (NA)	1011 (97.57)

Cite this article as: Fernandes Q, Augusto O, Chicumbe S, et al. Maternal and child health care service disruptions and recovery in Mozambique after Cyclone Idai: an uncontrolled interrupted time series analysis. *Glob Health Sci Pract*. 2022;10(Suppl 1):e2100796.

Table T4 - New Family Planning users

District/Province	2017 and 2018	March 2019	April 2019	Post
Overall	1860 (1889.78)	1676 (1333.22)	1627 (1336.43)	1699 (1728.78)
MANICA	2005 (2079.00)	1919 (1205.79)	2072 (1659.45)	2145 (1984.82)
BARUE	3479 (3699.19)	3311 (NA)	3220 (NA)	2566 (873.30)
CIDADE DE CHIMOIO	4283 (1927.24)	4701 (NA)	6251 (NA)	5707 (4032.09)
GONDOLA	2917 (2791.65)	2067 (NA)	1620 (NA)	2156 (780.62)
GURO	953 (816.88)	1012 (NA)	866 (NA)	1035 (294.06)
MACATE	1014 (833.18)	1032 (NA)	1104 (NA)	1698 (1792.14)
MACHAZE	1382 (696.89)	1398 (NA)	1535 (NA)	1404 (296.79)
MACOSSA	662 (347.05)	860 (NA)	824 (NA)	1092 (157.76)
MANICA	2046 (1035.70)	2132 (NA)	3161 (NA)	2504 (347.87)
MOSSURIZE	3022 (870.94)	2921 (NA)	3405 (NA)	3416 (1627.16)
SUSSUNDENGA	1916 (1559.12)	1744 (NA)	988 (NA)	2173 (1937.88)
TAMBARA	1149 (2237.49)	578 (NA)	499 (NA)	618 (75.62)
VANDUZI	1237 (1423.04)	1276 (NA)	1397 (NA)	1370 (1065.93)
SOFALA	1725 (1688.80)	1451 (1451.92)	1215 (815.91)	1287 (1332.30)
BUZI	2529 (1761.41)	644 (NA)	319 (NA)	1193 (692.63)
CAIA	2403 (2466.14)	5627 (NA)	1672 (NA)	1705 (1565.24)
CHEMBA	1129 (499.73)	571 (NA)	647 (NA)	658 (398.68)
CHERINGOMA	508 (433.46)	553 (NA)	557 (NA)	670 (641.78)
CHIBABAVA	891 (585.19)	696 (NA)	692 (NA)	907 (593.17)
CIDADE DA BEIRA	4386 (1902.22)	2597 (NA)	2241 (NA)	3052 (2540.09)
DONDO	1924 (1133.38)	758 (NA)	865 (NA)	1432 (929.97)
GORONGOSA	2167 (551.62)	2308 (NA)	2695 (NA)	1954 (965.20)
MACHANGA	539 (397.41)	434 (NA)	536 (NA)	640 (512.79)
MARINGUE	806 (631.58)	1166 (NA)	1312 (NA)	1005 (796.29)
MARROMEU	2081 (1481.50)	1724 (NA)	1839 (NA)	1200 (1327.03)
MUANZA	448 (328.12)	295 (NA)	284 (NA)	313 (170.53)
NHAMATANDA	2620 (2020.62)	1489 (NA)	2140 (NA)	2001 (1722.99)

Cite this article as: Fernandes Q, Augusto O, Chicumbe S, et al. Maternal and child health care service disruptions and recovery in Mozambique after Cyclone Idai: an uncontrolled interrupted time series analysis. *Glob Health Sci Pract*. 2022;10(Suppl 1):e2100796.

Table T5 - Number of Measles doses vaccination

District/Province	2017 and 2018	March 2019	April 2019	Post
Overall	598 (374.79)	565 (320.60)	532 (321.02)	638 (355.65)
MANICA	613 (359.88)	634 (306.09)	632 (321.18)	643 (307.88)
BARUE	832 (305.80)	981 (NA)	928 (NA)	1042 (78.84)
CIDADE DE CHIMOIO	1184 (303.27)	1116 (NA)	1126 (NA)	1193 (106.24)
GONDOLA	616 (209.77)	559 (NA)	619 (NA)	670 (67.20)
GURO	355 (81.91)	456 (NA)	419 (NA)	439 (68.84)
MACATE	433 (155.83)	362 (NA)	362 (NA)	392 (39.23)
MACHAZE	523 (191.87)	511 (NA)	497 (NA)	502 (26.47)
MACOSSA	186 (71.56)	228 (NA)	210 (NA)	233 (47.17)
MANICA	793 (231.50)	850 (NA)	826 (NA)	755 (72.93)
MOSSURIZE	1127 (191.53)	1062 (NA)	1101 (NA)	954 (176.64)
SUSSUNDENGA	595 (155.83)	694 (NA)	777 (NA)	762 (129.65)
TAMBARA	228 (79.99)	257 (NA)	184 (NA)	258 (59.79)
VANDUZI	479 (120.40)	529 (NA)	537 (NA)	519 (78.54)
SOFALA	584 (388.05)	502 (332.71)	440 (303.93)	634 (395.60)
BUZI	700 (160.34)	273 (NA)	75 (NA)	682 (91.58)
CAIA	677 (255.38)	764 (NA)	700 (NA)	741 (107.00)
CHEMBA	307 (90.67)	163 (NA)	217 (NA)	351 (183.78)
CHERINGOMA	270 (141.69)	279 (NA)	300 (NA)	318 (295.83)
CHIBABAVA	554 (91.55)	533 (NA)	486 (NA)	537 (93.07)
CIDADE DA BEIRA	1511 (310.70)	1255 (NA)	1206 (NA)	1572 (129.39)
DONDO	619 (102.01)	354 (NA)	442 (NA)	665 (45.69)
GORONGOSA	606 (56.43)	582 (NA)	621 (NA)	679 (85.05)
MACHANGA	232 (50.13)	205 (NA)	172 (NA)	252 (77.08)
MARINGUE	340 (132.23)	419 (NA)	316 (NA)	372 (73.64)
MARROMEU	571 (99.92)	702 (NA)	563 (NA)	720 (155.52)
MUANZA	148 (54.53)	106 (NA)	110 (NA)	170 (50.22)
NHAMATANDA	1056 (196.11)	895 (NA)	514 (NA)	1176 (101.35)

Cite this article as: Fernandes Q, Augusto O, Chicumbe S, et al. Maternal and child health care service disruptions and recovery in Mozambique after Cyclone Idai: an uncontrolled interrupted time series analysis. *Glob Health Sci Pract*. 2022;10(Suppl 1):e2100796.

Table T6 - Number of BCG doses vaccination

District/Province	2017 and 2018	March 2019	April 2019	Post
Overall	669 (417.30)	591 (352.29)	646 (404.09)	712 (413.16)
MANICA	692 (422.15)	676 (357.94)	745 (412.32)	750 (398.89)
BARUE	942 (211.99)	991 (NA)	992 (NA)	1067 (72.61)
CIDADE DE CHIMOIO	1497 (176.96)	1415 (NA)	1543 (NA)	1575 (62.02)
GONDOLA	699 (119.19)	737 (NA)	749 (NA)	859 (85.41)
GURO	408 (49.81)	429 (NA)	470 (NA)	467 (48.20)
MACATE	443 (143.47)	360 (NA)	387 (NA)	407 (31.58)
MACHAZE	611 (390.34)	549 (NA)	576 (NA)	556 (44.13)
MACOSSA	175 (32.09)	221 (NA)	205 (NA)	239 (15.16)
MANICA	837 (106.77)	846 (NA)	985 (NA)	1073 (286.70)
MOSSURIZE	1144 (542.92)	1085 (NA)	1339 (NA)	1059 (175.02)
SUSSUNDENGA	720 (110.77)	682 (NA)	810 (NA)	830 (115.86)
TAMBARA	248 (50.97)	289 (NA)	282 (NA)	262 (54.74)
VANDUZI	577 (63.37)	502 (NA)	606 (NA)	609 (57.32)
SOFALA	648 (412.37)	512 (341.99)	554 (389.57)	676 (424.09)
BUZI	712 (152.79)	252 (NA)	66 (NA)	743 (136.74)
CAIA	779 (196.58)	667 (NA)	771 (NA)	746 (103.39)
CHEMBA	346 (66.24)	198 (NA)	324 (NA)	371 (181.35)
CHERINGOMA	307 (99.97)	258 (NA)	315 (NA)	311 (118.31)
CHIBABAVA	588 (102.04)	547 (NA)	563 (NA)	603 (78.52)
CIDADE DA BEIRA	1650 (232.86)	1283 (NA)	1536 (NA)	1704 (278.44)
DONDO	656 (183.27)	302 (NA)	545 (NA)	657 (80.96)
GORONGOSA	796 (131.49)	747 (NA)	832 (NA)	833 (106.11)
MACHANGA	245 (27.55)	230 (NA)	185 (NA)	250 (37.28)
MARINGUE	396 (81.61)	464 (NA)	574 (NA)	448 (82.12)
MARROMEU	657 (67.43)	643 (NA)	695 (NA)	748 (165.68)
MUANZA	150 (45.53)	115 (NA)	100 (NA)	166 (42.98)
NHAMATANDA	1148 (161.67)	956 (NA)	701 (NA)	1215 (121.84)

Cite this article as: Fernandes Q, Augusto O, Chicumbe S, et al. Maternal and child health care service disruptions and recovery in Mozambique after Cyclone Idai: an uncontrolled interrupted time series analysis. *Glob Health Sci Pract*. 2022;10(Suppl 1):e2100796.

Table T7 - Number of DPT-Hib 3 doses vaccination

District/Province	2017 and 2018	March 2019	April 2019	Post
Overall	606 (360.39)	578 (325.11)	526 (317.93)	640 (354.59)
MANICA	615 (336.32)	672 (340.83)	648 (333.80)	652 (324.46)
BARUE	848 (263.48)	951 (NA)	1000 (NA)	1066 (70.73)
CIDADE DE CHIMOIO	1199 (144.41)	1344 (NA)	1181 (NA)	1268 (99.17)
GONDOLA	609 (139.83)	683 (NA)	681 (NA)	671 (88.32)
GURO	366 (48.91)	506 (NA)	425 (NA)	437 (38.32)
MACATE	420 (151.56)	343 (NA)	343 (NA)	377 (28.44)
MACHAZE	512 (126.10)	504 (NA)	477 (NA)	508 (33.60)
MACOSSA	168 (19.83)	217 (NA)	197 (NA)	244 (23.23)
MANICA	799 (101.42)	855 (NA)	862 (NA)	787 (93.40)
MOSSURIZE	1102 (200.44)	1096 (NA)	1053 (NA)	971 (160.58)
SUSSUNDENGA	632 (109.56)	638 (NA)	812 (NA)	759 (102.59)
TAMBARA	234 (72.84)	269 (NA)	211 (NA)	250 (49.43)
VANDUZI	494 (69.72)	653 (NA)	532 (NA)	488 (88.99)
SOFALA	597 (381.59)	492 (296.78)	413 (267.29)	630 (381.02)
BUZI	746 (176.29)	331 (NA)	47 (NA)	692 (113.85)
CAIA	713 (267.61)	735 (NA)	735 (NA)	728 (94.21)
CHEMBA	300 (67.87)	138 (NA)	232 (NA)	345 (121.45)
CHERINGOMA	283 (110.72)	252 (NA)	252 (NA)	288 (92.77)
CHIBABAVA	557 (78.07)	637 (NA)	504 (NA)	551 (67.16)
CIDADE DA BEIRA	1504 (144.35)	1065 (NA)	951 (NA)	1547 (175.11)
DONDO	661 (59.92)	396 (NA)	404 (NA)	671 (77.86)
GORONGOSA	619 (67.97)	621 (NA)	597 (NA)	716 (69.03)
MACHANGA	230 (31.72)	214 (NA)	189 (NA)	252 (53.58)
MARINGUE	333 (119.47)	399 (NA)	242 (NA)	399 (70.11)
MARROMEU	628 (107.64)	624 (NA)	567 (NA)	704 (122.91)
MUANZA	139 (39.80)	96 (NA)	91 (NA)	151 (35.07)
NHAMATANDA	1053 (195.34)	884 (NA)	563 (NA)	1141 (103.88)

Cite this article as: Fernandes Q, Augusto O, Chicumbe S, et al. Maternal and child health care service disruptions and recovery in Mozambique after Cyclone Idai: an uncontrolled interrupted time series analysis. *Glob Health Sci Pract*. 2022;10(Suppl 1):e2100796.

Table T8 - Fully immunized children underage of 1 year

District/Province	2017 and 2018	March 2019	April 2019	Post
Overall	546 (352.55)	532 (333.66)	503 (306.69)	595 (342.68)
MANICA	581 (358.20)	592 (305.37)	607 (305.69)	621 (300.82)
BARUE	824 (472.68)	918 (NA)	900 (NA)	1021 (77.39)
CIDADE DE CHIMOIO	1104 (171.56)	1116 (NA)	1126 (NA)	1182 (104.92)
GONDOLA	574 (206.88)	578 (NA)	605 (NA)	648 (76.45)
GURO	332 (75.50)	437 (NA)	414 (NA)	428 (62.25)
MACATE	422 (153.90)	355 (NA)	356 (NA)	408 (93.47)
MACHAZE	495 (163.00)	485 (NA)	492 (NA)	486 (22.90)
MACOSSA	233 (311.56)	225 (NA)	190 (NA)	234 (47.48)
MANICA	733 (254.90)	812 (NA)	747 (NA)	699 (69.94)
MOSSURIZE	1054 (180.17)	1035 (NA)	1045 (NA)	906 (162.21)
SUSSUNDENGA	566 (159.16)	388 (NA)	678 (NA)	718 (114.35)
TAMBARA	191 (70.84)	237 (NA)	203 (NA)	228 (53.08)
VANDUZI	442 (109.10)	514 (NA)	532 (NA)	493 (76.44)
SOFALA	514 (344.68)	477 (360.99)	406 (285.33)	570 (376.54)
BUZI	640 (121.25)	243 (NA)	66 (NA)	653 (94.43)
CAIA	603 (191.91)	703 (NA)	619 (NA)	675 (120.92)
CHEMBA	257 (75.73)	139 (NA)	194 (NA)	268 (135.85)
CHERINGOMA	241 (131.67)	293 (NA)	262 (NA)	228 (120.43)
CHIBABAVA	474 (67.40)	479 (NA)	456 (NA)	502 (50.99)
CIDADE DA BEIRA	1315 (385.16)	1421 (NA)	1131 (NA)	1491 (181.65)
DONDO	584 (91.92)	296 (NA)	427 (NA)	624 (55.24)
GORONGOSA	537 (53.99)	565 (NA)	579 (NA)	617 (74.80)
MACHANGA	217 (48.98)	197 (NA)	157 (NA)	223 (29.42)
MARINGUE	254 (94.49)	336 (NA)	267 (NA)	308 (62.42)
MARROMEU	534 (123.42)	586 (NA)	523 (NA)	608 (144.59)
MUANZA	131 (50.02)	104 (NA)	107 (NA)	146 (40.04)
NHAMATANDA	894 (156.70)	836 (NA)	492 (NA)	1069 (107.20)

Cite this article as: Fernandes Q, Augusto O, Chicumbe S, et al. Maternal and child health care service disruptions and recovery in Mozambique after Cyclone Idai: an uncontrolled interrupted time series analysis. *Glob Health Sci Pract*. 2022;10(Suppl 1):e2100796.

Table T9 - First at-risk children's consultation

District/Province	2017 and 2018	March 2019	April 2019	Post
Overall	101 (117.57)	92 (88.18)	101 (105.42)	131 (115.69)
MANICA	77 (55.92)	86 (60.82)	95 (69.03)	122 (71.87)
BARUE	97 (28.05)	93 (NA)	75 (NA)	168 (42.51)
CIDADE DE CHIMOIO	219 (29.45)	251 (NA)	252 (NA)	284 (25.63)
GONDOLA	83 (23.07)	85 (NA)	98 (NA)	125 (24.67)
GURO	50 (21.04)	80 (NA)	86 (NA)	70 (12.70)
MACATE	44 (10.74)	23 (NA)	63 (NA)	62 (19.77)
MACHAZE	51 (13.43)	52 (NA)	72 (NA)	90 (28.12)
MACOSSA	19 (14.69)	40 (NA)	44 (NA)	63 (16.19)
MANICA	122 (18.66)	138 (NA)	223 (NA)	202 (40.56)
MOSSURIZE	54 (16.62)	78 (NA)	69 (NA)	136 (36.29)
SUSSUNDENGA	67 (19.33)	76 (NA)	53 (NA)	117 (18.96)
TAMBARA	19 (5.07)	29 (NA)	34 (NA)	38 (17.93)
VANDUZI	57 (14.42)	81 (NA)	71 (NA)	110 (34.61)
SOFALA	122 (148.61)	99 (111.61)	107 (133.36)	140 (144.55)
BUZI	124 (34.74)	0 (NA)	1 (NA)	142 (23.51)
CAIA	144 (32.50)	146 (NA)	165 (NA)	185 (29.09)
CHEMBA	39 (27.08)	33 (NA)	45 (NA)	63 (11.30)
CHERINGOMA	18 (7.61)	14 (NA)	15 (NA)	20 (6.63)
CHIBABAVA	84 (18.98)	66 (NA)	53 (NA)	90 (23.26)
CIDADE DA BEIRA	581 (111.68)	422 (NA)	500 (NA)	558 (100.24)
DONDO	141 (28.71)	120 (NA)	132 (NA)	195 (17.81)
GORONGOSA	159 (69.26)	126 (NA)	188 (NA)	188 (68.94)
MACHANGA	41 (13.68)	27 (NA)	30 (NA)	30 (5.07)
MARINGUE	42 (10.89)	62 (NA)	60 (NA)	63 (16.60)
MARROMEU	58 (16.19)	46 (NA)	54 (NA)	59 (10.52)
MUANZA	9 (3.75)	15 (NA)	7 (NA)	14 (8.89)
NHAMATANDA	151 (26.34)	114 (NA)	139 (NA)	212 (54.54)

Cite this article as: Fernandes Q, Augusto O, Chicumbe S, et al. Maternal and child health care service disruptions and recovery in Mozambique after Cyclone Idai: an uncontrolled interrupted time series analysis. *Glob Health Sci Pract*. 2022;10(Suppl 1):e2100796.

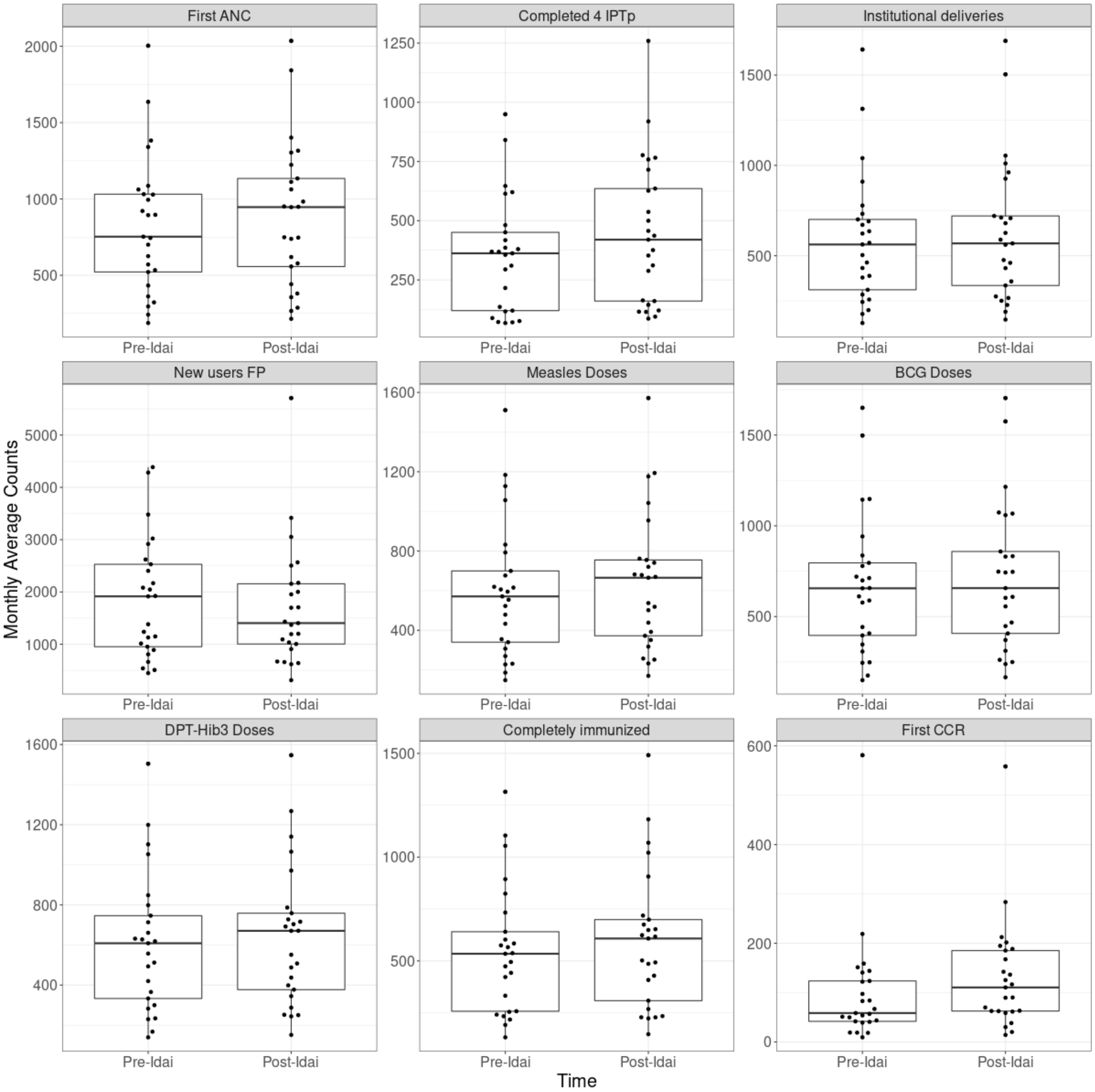

Figure T1 – District level comparison of average service provision counts for pre- and post- Idai. Pre- includes (2017 and 2018 months) and post-Idai

Cite this article as: Fernandes Q, Augusto O, Chicumbe S, et al. Maternal and child health care service disruptions and recovery in Mozambique after Cyclone Idai: an uncontrolled interrupted time series analysis. *Glob Health Sci Pract*. 2022;10(Suppl 1):e2100796.

Average counts per district strata of affectedness

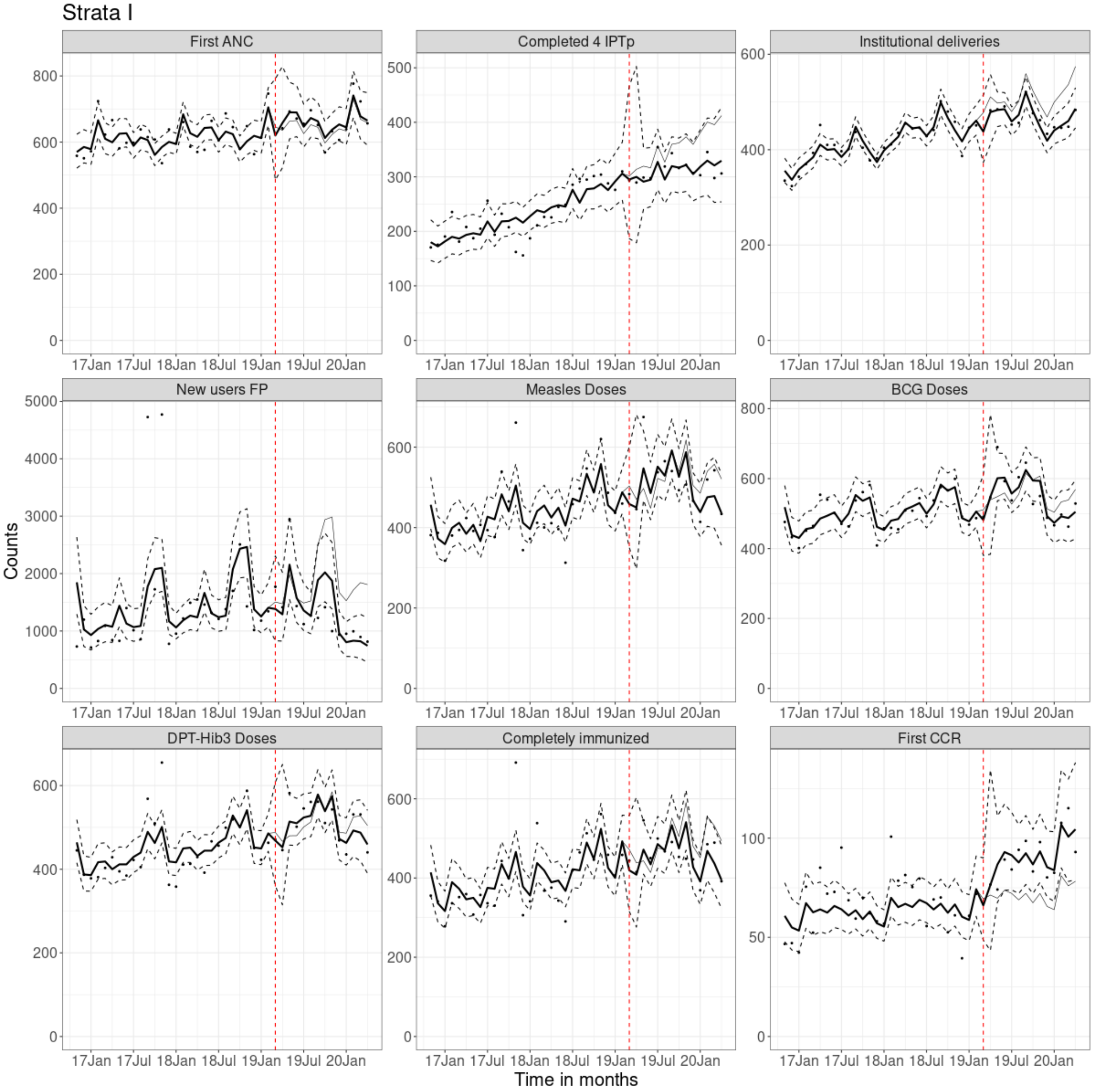

Figure U1 – The strata I (lower Idai Cyclone affectedness) comparison of average service provision counts for pre- and post- Idai. Pre- includes (2017 and 2018 months) and post-Idai

Cite this article as: Fernandes Q, Augusto O, Chicumbe S, et al. Maternal and child health care service disruptions and recovery in Mozambique after Cyclone Idai: an uncontrolled interrupted time series analysis. *Glob Health Sci Pract*. 2022;10(Suppl 1):e2100796.

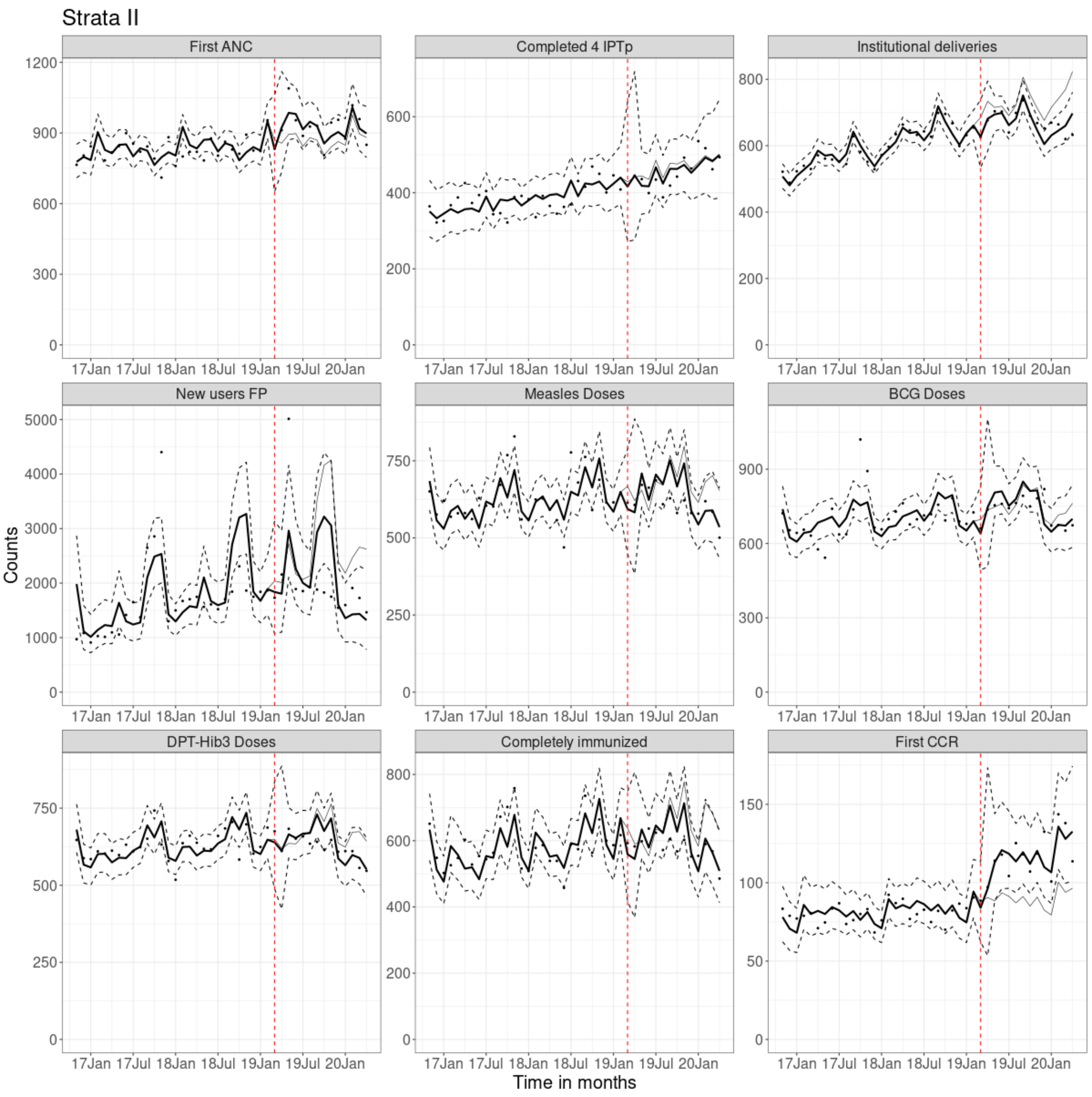

Figure U2 – The strata II (moderately Idai Cyclone affectedness) comparison of average service provision counts for pre- and post- Idai. Pre- includes (2017 and 2018 months) and post-Idai

Cite this article as: Fernandes Q, Augusto O, Chicumbe S, et al. Maternal and child health care service disruptions and recovery in Mozambique after Cyclone Idai: an uncontrolled interrupted time series analysis. *Glob Health Sci Pract*. 2022;10(Suppl 1):e2100796.

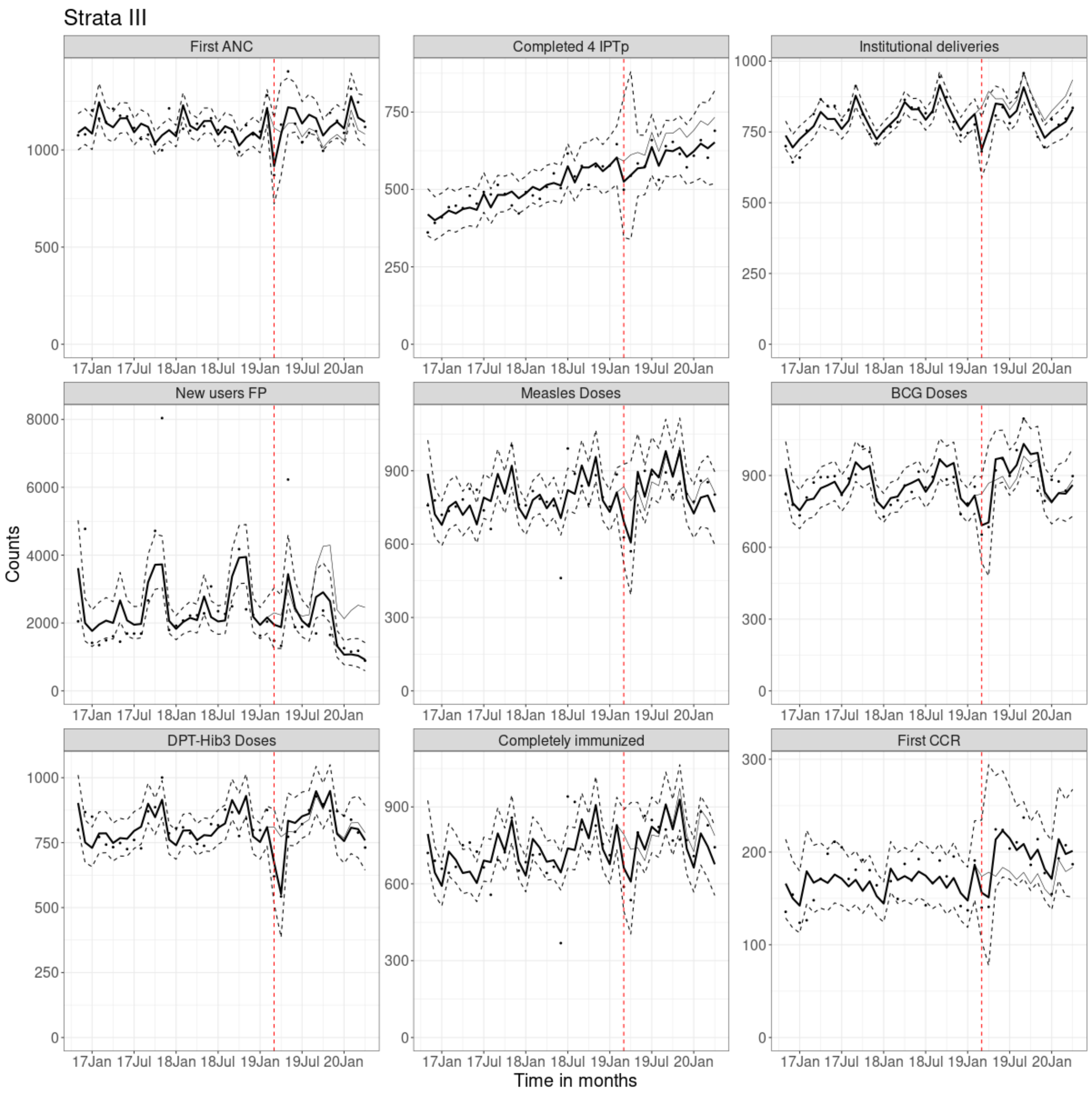

Figure U3 – The strata III (highly Idai Cyclone affectedness) comparison of average service provision counts for pre- and post- Idai. Pre- includes (2017 and 2018 months) and post-Idai

Cite this article as: Fernandes Q, Augusto O, Chicumbe S, et al. Maternal and child health care service disruptions and recovery in Mozambique after Cyclone Idai: an uncontrolled interrupted time series analysis. *Glob Health Sci Pract*. 2022;10(Suppl 1):e2100796.

Negative binomial regression with random-intercepts (per reviewer request)

Table V1 - Negative Binomial with random-intercepts and March and April 2019 coded as one dummy indicator

Indicator	Intercept	Post	Pre-slope	Post-slope change
First ANC	948.3 (847.7; 1060.9)	1.090 (1.031; 1.152)	0.999 (0.998; 1.001)	0.995 (0.989; 1.002)
Completed 4 IPTp	273.8 (207.5; 361.4)	0.945 (0.848; 1.052)	1.012 (1.009; 1.015)	0.999 (0.987; 1.012)
Institutional deliveries	568.4 (513.6; 628.9)	1.003 (0.962; 1.045)	1.005 (1.004; 1.006)	0.988 (0.983; 0.993)
New users of FP	1501.9 (1255.4; 1796.7)	1.313 (1.089; 1.585)	1.013 (1.008; 1.019)	0.918 (0.899; 0.938)
Measles doses	617.6 (561.9; 678.8)	1.171 (1.070; 1.283)	1.004 (1.001; 1.006)	0.974 (0.964; 0.985)
BCG doeses	717.5 (652.8; 788.6)	1.137 (1.054; 1.227)	1.001 (0.999; 1.003)	0.984 (0.975; 0.993)
DPT-Hib3 doses	638.2 (582.9; 698.7)	1.100 (1.021; 1.184)	1.002 (1.000; 1.004)	0.985 (0.977; 0.994)
Completely immunized	553.1 (494.8; 618.4)	1.137 (1.029; 1.256)	1.005 (1.002; 1.008)	0.973 (0.962; 0.985)
First CCR	79.6 (60.6; 104.5)	1.297 (1.142; 1.473)	1.001 (0.998; 1.005)	1.007 (0.992; 1.022)

Indicator	Strata II	Strata III	MarchApril 2019	Interaction: Strata II and MarchApril2019	Interaction: Strata III and MarchApril2019
First ANC	1.012 (0.856; 1.198)	0.963 (0.820; 1.131)	0.927 (0.844; 1.017)	0.983 (0.880; 1.097)	0.852 (0.766; 0.948)
Completed 4 IPTp	1.175 (0.769; 1.796)	1.195 (0.797; 1.793)	0.984 (0.822; 1.179)	1.132 (0.914; 1.403)	0.803 (0.653; 0.986)
Institutional deliveries	1.030 (0.883; 1.202)	0.945 (0.815; 1.095)	0.932 (0.870; 1.000)	1.000 (0.921; 1.085)	0.829 (0.765; 0.897)
New users of FP	0.867 (0.701; 1.073)	0.944 (0.771; 1.157)	0.812 (0.595; 1.108)	0.892 (0.617; 1.291)	0.567 (0.398; 0.807)
Measles doses	1.032 (0.919; 1.160)	0.945 (0.845; 1.057)	0.844 (0.725; 0.982)	0.973 (0.812; 1.165)	0.732 (0.615; 0.871)
BCG doeses	1.020 (0.897; 1.158)	0.908 (0.803; 1.026)	0.892 (0.786; 1.014)	0.965 (0.830; 1.123)	0.732 (0.633; 0.848)
DPT-Hib3 doses	1.022 (0.905; 1.155)	0.937 (0.834; 1.053)	0.870 (0.769; 0.984)	1.062 (0.917; 1.230)	0.769 (0.667; 0.886)
Completely immunized	1.058 (0.916; 1.221)	0.969 (0.845; 1.112)	0.854 (0.723; 1.010)	0.984 (0.807; 1.200)	0.708 (0.585; 0.857)
First CCR	0.973 (0.646; 1.466)	0.946 (0.640; 1.400)	0.825 (0.668; 1.020)	0.931 (0.726; 1.196)	0.755 (0.588; 0.970)

Cite this article as: Fernandes Q, Augusto O, Chicumbe S, et al. Maternal and child health care service disruptions and recovery in Mozambique after Cyclone Idai: an uncontrolled interrupted time series analysis. *Glob Health Sci Pract*. 2022;10(Suppl 1):e2100796.

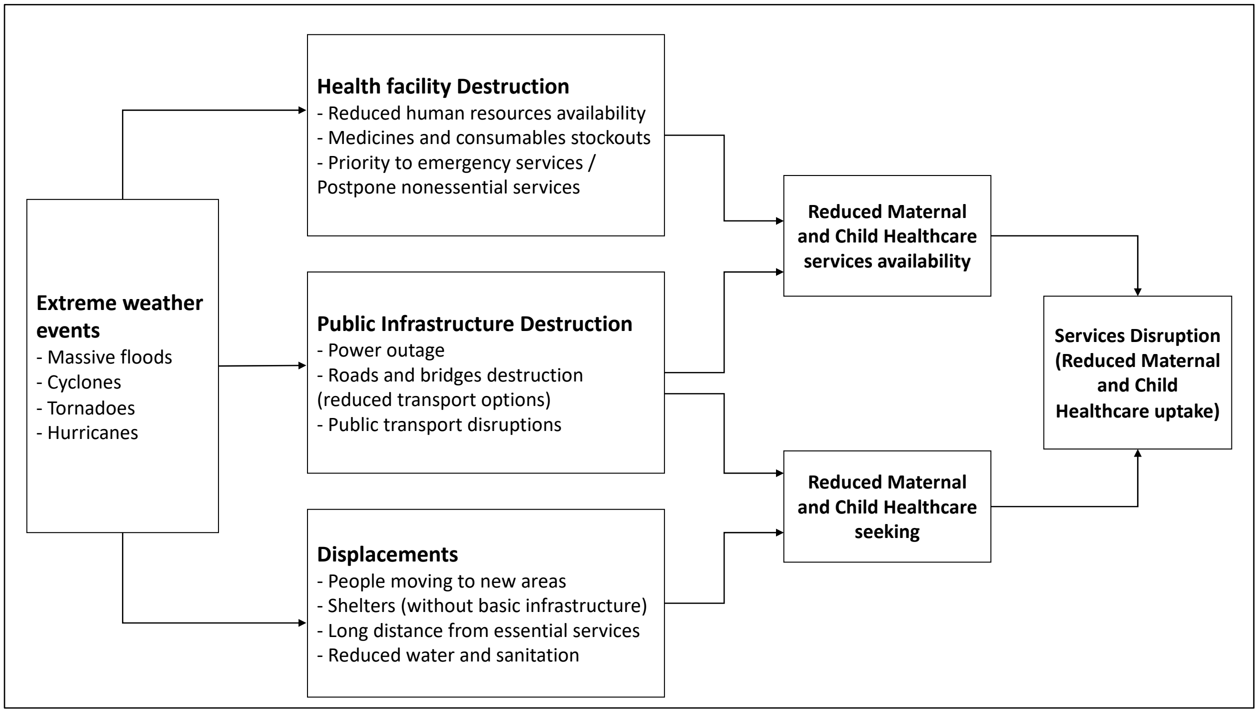

Figure Z1 – Conceptual framework

Negative impacts on public infrastructure from extreme weather events are expected. Immediate consequences lead to public infrastructure destruction, people displacements, and changes in healthcare-seeking dynamics, leading to reduced accessibility, availability, and quality of healthcare services. Maternal and child healthcare services may also be affected due to reduced services and reduced health care seeking. The conceptual framework summarizes how the IDAI cyclone could have affected Maternal and Child Healthcare in Manica and Sofala provinces.
